# Maternal Aspartame Exposure Induces Neonatal Pulmonary Metabolic Dysregulation and Redox Imbalance: A Multiomics Investigation of Gut Microbiota-Host Interactions

**DOI:** 10.1021/acs.jafc.5c08819

**Published:** 2025-10-13

**Authors:** Sheng-Yuan Ho, Cheng-Yang Lee, Hsiu-Chu Chou, Liang-Ti Huang, Chung-Ming Chen

**Affiliations:** † Graduate Institute of Clinical Medicine, College of Medicine, Taipei Medical University, Taipei 11031, Taiwan; ‡ Department of Pediatrics, Tri-Service General Hospital, National Defense Medical University, Taipei 11490, Taiwan; § Department of Pediatrics, School of Medicine, College of Medicine, National Defense Medical University, Taipei 11031, Taiwan; ∥ Bioinformatics Center, Office of Data Science, Taipei Medical University, Taipei 11031, Taiwan; ⊥ Department of Anatomy and Cell Biology, School of Medicine, College of Medicine, Taipei Medical University, Taipei 11031, Taiwan; # Department of Pediatrics, Wan Fang Hospital, Taipei Medical University, Taipei 11696, Taiwan; ∇ Department of Pediatrics, School of Medicine, College of Medicine, Taipei Medical University, Taipei 11031, Taiwan; ○ Department of Pediatrics, Taipei Medical University Hospital, Taipei 11031, Taiwan; ◆ TMU Research Center for Digestive Medicine, Taipei Medical University, Taipei 11031, Taiwan

**Keywords:** aspartame, gut-lung axis, gut microbiota, metabolic dysregulation, inflammasome, oxidative stress

## Abstract

Redox imbalance during development may make offspring more vulnerable to long-term pulmonary issues. Maternal aspartame intake was investigated for its influence on neonatal lung redox biology. Using a multiomics approach (untargeted metabolomics, gut microbiota profiling, redox-inflammation markers), aspartame exposure (0.25 g/L in drinking water from gestational day 7 to postnatal day 21) was found to significantly alter neonatal pulmonary metabolic profiles, especially purine metabolism and the pentose phosphate pathway. MetOrigin analysis suggested a potential link between these metabolic changes and host-microbiota cometabolism. Elevated 8-hydroxy-2′-deoxyguanosine and malondialdehyde, along with decreased glutathione, suggested oxidative stress. These redox disturbances occurred with increased cleaved caspase-1 and interleukin-1β, consistent with inflammasome activation. Taken together, these integrated chemical and biological data propose a potential pathway by which early life aspartame exposures may disrupt redox-sensitive metabolic networks and inflammatory responses in the developing lung. These results emphasize the value of assessing artificial sweeteners when considering developmental redox homeostasis and immune–metabolic health.

## Graphical abstract

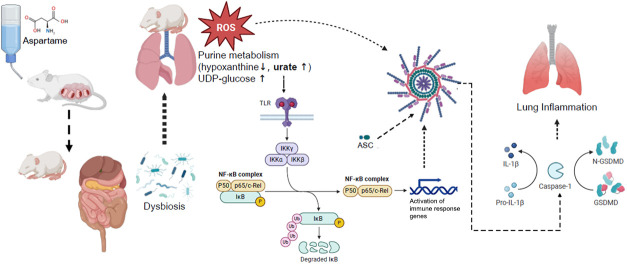

## 1 Introduction

The widespread consumption of artificial sweeteners, especially aspartame, has raised increasing concerns regarding their long-term metabolic implications, above all during important developmental windows like pregnancy. As a growing area of focus in redox biology, the impact of dietary exposures on oxidative homeostasis and inflammation during fetal and neonatal periods warrants close attention. Emerging evidence suggests that maternal aspartame intake increases offspring’s likelihood of allergic diseases, such as asthma,^1,2^ which often involve underlying metabolic dysregulation.^3^ Asthma is characterized by chronic airway inflammation driven by a T-helper cell type 2 (Th2)-biased immune response.^4^ However, the upstream mechanisms linking maternal aspartame exposure to immune dysregulation, particularly those involving fundamental metabolic alterations and cellular redox status, in offspring remain poorly understood. While numerous studies have investigated the long-term metabolic effects of various artificial sweeteners in adult models, often focusing on systemic parameters like body weight and glucose metabolism,^5^ the key question of their impact during vulnerable developmental windows, such as pregnancy, on offspring organ-specific metabolic programming remains largely unaddressed. Specifically, the consequences of maternal artificial sweetener exposure on neonatal lung metabolism and its susceptibility to later-life diseases are poorly understood.

Recent research highlights the gut-lung axis as a central contributor to asthma pathogenesis and systemic metabolic health.^6^ Gut microbiota dysbiosis can worsen lung inflammation through disrupted gut-derived metabolites, impaired intestinal barrier integrity, and immune signaling, chiefly via the Toll-like receptor 4/Nuclear factor kappa-light-chain-enhancer of activated B cells (NF-κB) pathway.^7,8^ These observations suggest that maternal dietary factors, such as aspartame, may influence the gut-lung axis, potentially disrupting metabolic homeostasis and cellular redox balance in offspring, thereby contributing to disease vulnerability.

Emerging evidence indicates that sex is a critical biological variable in studies of developmental metabolism and immune programming, as males and females often exhibit distinct responses to environmental stressors.^9^ In this context, metabolic dysregulation is closely linked to oxidative stress and inflammasome activation. Specifically, the NLR family pyrin domain containing 3 (NLRP3) inflammasome has emerged as a main mediator of metabolic dysregulation and Th2-driven inflammation.^10,11^ NLRP3 acts as a sensor of danger-associated molecular patterns (DAMPs) and reactive oxygen species (ROS), triggering inflammasome assembly and interleukin (IL)-1β production.^12,13^ Given that metabolic stressors and NF-κB signaling are upstream activators of NLRP3, and often operate together with redox perturbations, it is reasonable to hypothesize that maternal aspartame exposure induces oxidative stress and compromises antioxidant defenses, consequently leading to inflammasome activation in offspring and contributing to lung inflammation.^14–16^

Our previous study using the same maternal aspartame exposure model and animal cohort showed that maternal aspartame intake activates NF-κB signaling, elevates serum immunoglobulin E levels, and promotes a Th2-skewed immune response in offspring, alongside alterations in gut microbiota composition.^17^ Building on these results, the direct role of lung metabolic disruptions, detailed oxidative stress profiles, and the subsequent activation of the NLRP3 inflammasome in the lung, remains largely unexplored.

This study hypothesizes that maternal aspartame consumption disrupts neonatal lung metabolism and redox homeostasis, with these effects being associated with alterations in the gut-lung axis, which could result in oxidative stress and inflammasome activation. Combining untargeted lung metabolomics to identify precise biochemical alterations and previously published gut microbiota data,^17^ this investigation explores the intricate connection between microbial shifts and host metabolic adaptations that may collectively contribute to oxidative stress and pulmonary inflammation in the offspring. This biochemistry-driven approach deepens our molecular knowledge of how early life dietary exposures reprogram immune and metabolic networks through redox-sensitive pathways.

## 2 Materials and Methods

### 2.1 Animal Model

All experimental procedures received approval from the Taipei Medical University Animal Care and Use Committee and followed institutional guidelines (IACUC approval number: LAC2023-0108). The animal model, including maternal aspartame exposure and offspring maintenance, was established and conducted as previously described by Chuang et al.^17^ Briefly, pregnant BALB/c mice were randomly assigned to a control group and an aspartame group. Aspartame (0.25 g/L) was administered through drinking water from gestational day 7 until postnatal day 21. This dose (40 mg/kg/day in mice) represents a physiologically relevant upper-range exposure. Using allometric scaling, this corresponds to a human equivalent dose of ∼3.25 mg/kg/day (∼195 mg/day for a 60 kg adult), which is comparable to the aspartame content of approximately one can of diet soda. Thus, the chosen dose reflects high but realistic consumption levels while remaining well below the FDA acceptable daily intake (detailed calculations in Supporting Methods).^17,18^

### 2.2 Tissue Collection and Preparation

On PND 21, lung and gastrointestinal tissues were harvested after euthanasia and stored at −80 °C. For microbiota analyses, luminal contents were collected by gently expressing fecal pellets from the colon immediately after dissection. The collected material was transferred into sterile tubes using ethanol- and heat-sterilized forceps, snap-frozen in liquid nitrogen, and stored at −80 °C until DNA extraction. Tissue processing and microbial DNA extraction followed protocols previously described.^17^ For this study, lung tissues were processed for metabolomic and oxidative stress analyses, as detailed below. To obtain samples for the metabolomics and ELISA assays (*n* = 14 per group), pups were selected from 5 control dams and 3 aspartame-exposed dams, with representation across litters and balanced distribution of males and females.

### 2.3 Preparation of Samples for Metabolomic Analysis

We prepared lung tissue extracts in 100 μL of methanol (Macron Chemicals, Center Valley, PA)–water (Sigma-Aldrich, St. Louis, MO) (7:3, v/v). After two freeze–thaw cycles and vortexing, the samples were centrifuged at 12,000*g* for 15 min at 4 °C. The resulting supernatants were dried under vacuum and reconstituted in 0.3 mL of a water–acetonitrile mixture (50:50, v/v). A pooled quality-control sample was generated by combining equal volumes of individual supernatants.

### 2.4 Metabolomic Analysis via Liquid Chromatography and Mass Spectrometry

Metabolomic profiling was performed using an ACQUITY UPLC system coupled with a SYNAPT G2 quadrupole time-of-flight mass spectrometer (Waters, UK). Chromatographic separation utilized a BEH C18 column (2.1 × 100 mm, 1.7 μm) at 45 °C, with a mobile phase gradient of water (A) and acetonitrile with 0.1% formic acid (B). Key mass spectrometry (MS) parameters included a source temperature of 120 °C, desolvation gas flow of 900 L/h at 550 °C, cone gas flow of 15 L/h, capillary voltage of 2.8 kV, and cone voltage of 40 V. Data acquisition was conducted in negative ion mode over an *m*/*z* range of 50–1000, using MassLynx (version 4.1). Leucine-enkephalin (*m*/*z* = 556.2771) served as a lockmass standard, infused at 5 μL/min with lockspray intervals of 20 s.

### 2.5 Data Processing and Pathway Analysis

We processed raw MS data using Progenesis QI software (Waters) for peak detection, alignment, and normalization. Metabolites with ≥2.0-fold differences in median intensity were considered significant. We identified metabolites based on mass accuracy (<5 ppm), isotope patterns, and spectral matching against HMDB, Metlin, and MassBank. A compound prediction threshold score of ≥36 was applied. Quality control was ensured using pooled reference samples, achieving ≥90% alignment with chromatographic profiles. Retention time and *m*/*z* pairs were aggregated using adduct and isotope deconvolution methods to ensure reliable ion abundance calculations. For metabolomics data, log10 transformation and scalar adjustments were applied to normalize distributions. Significant metabolic features were visualized using volcano plots. Dimensionality reduction and clustering analyses were performed using principal component analysis (PCA) and partial least-squares discriminant analysis (PLS-DA), with key metabolites identified by variable importance in projection (VIP) scores. PLS-DA model performance was assessed via 10-fold cross-validation, evaluating *R* ^*2*^ and *Q* ^*2*^ metrics for up to eight components. Model robustness was further examined using a 2000-permutation test based on the separation distance (B/W). Pathway analysis was performed with MetaboAnalyst 6.0, mapping identified compounds to the Human Metabolome Database (HMDB) and KEGG pathways. Enrichment analysis used Fisher’s exact test with false discovery rate (FDR) correction (FDR < 0.05). To ensure biological relevance and annotation confidence, manual curation of metabolomic features was performed prior to pathway mapping. Metabolites were excluded if they were classified as exogenous compounds (e.g., drugs, environmental chemicals) based on HMDB and KEGG cross-referencing, or had low identification scores (<36).

### 2.6 Western Blot Analysis

For Western blot analysis, a subset of samples (*n* = 8 per group) was selected from 4 control dams and 3 aspartame-exposed dams, ensuring balanced representation of sexes and litters. This sample size allowed all replicates for a given protein to be run on a single gel, thereby minimizing intergel batch effects. Lung tissues underwent trypsinization, were subsequently washed with phosphate-buffered saline (PBS), and then centrifuged at 1500 rpm for 7 min. Next, 100 μL of lysis solution and a protease inhibitor were added. Following a 30 min incubation on ice, the samples were centrifuged in a microcentrifuge at 12,000 rpm for 20 min at 4 °C. The supernatant was carefully transferred to a separate prechilled tube on ice, while the pellet was discarded. Proteins (30 μg) were separated via sodium dodecyl sulfate-polyacrylamide gel electrophoresis (SDS-PAGE), using a 12% polyacrylamide gel for Apoptosis-associated Speck-like protein containing a CARD (ASC), a 16% gel for IL-1β, and a 10% gel for cleaved caspase-1. Separated proteins were subsequently transferred onto polyvinylidene difluoride membranes (ImmobilonP, Millipore, Bedford, MA) through electroblotting. After this, the membranes were subjected to blocking with 5% nonfat dried milk and incubated with primary antibodies targeting cleaved caspase-1 (1:750, Thermo Fisher Scientific, Waltham, MA), ASC (1:500, Bioss Inc., Woburn, MA), and IL-1β (1:750, Abcam, Cambridge, UK). For loading controls, β-actin antibody was used for ASC and IL-1β blots, while GAPDH antibody was used for cleaved caspase-1 blots. Following primary antibody incubation, membranes were treated with a horseradish peroxidase-conjugated goat antimouse antibody (Pierce Biotechnology, Rockford, IL). Finally, protein bands were visualized with the BioSpectrum AC Imaging System (UVP, Upland, California). Protein expression levels were quantified using ImageJ software and normalized to their respective loading controls on the same membrane.

### 2.7 Enzyme-Linked Immunosorbent Assay

We quantified lung 8-hydroxy-2′-deoxyguanosine (8-OHdG), malondialdehyde (MDA), and glutathione (GSH) levels using mouse ELISA kits (MyBioSource, San Diego, CA) following the manufacturer’s protocols. Each assay was validated with a standard curve using the supplied calibrators. The calibration curves showed excellent linearity within the tested ranges (GSH: *y* = 0.0121*x* + 0.1796, *R* ^2^ = 0.9905; MDA: *y* = 0.0247*x* + 0.2029, *R* ^2^ = 0.9908; 8-OHdG: *y* = 0.1186*x* + 0.068, *R* ^2^ = 0.9986). Representative raw optical density values are provided in Supporting Table S1.

### 2.8 16S rDNA Sequencing for Gut Microbiota

Gut microbiota 16S rDNA sequencing data utilized for correlation analyses in this study were obtained from the same animal cohort, with detailed methods for genomic DNA extraction from colon fecal samples and lung tissue, sequencing on the Illumina MiSeq platform, and subsequent analysis previously described in Chuang et al.^17^ The sequence reads have been deposited in the European Nucleotide Archive under accession number PRJEB28574.

### 2.9 Analysis of Metabolite Origins and Enriched Pathways

We employed the MetOrigin 2.0 platform to conduct a more thorough analysis of metabolites found through untargeted metabolomics. Using the Deep MetOrigin Analysis mode on its publicly available online interface (http://metorigin.met-bioinformatics.cn/),^19^ we classified metabolites according to their origins—designated as host-derived, microbiota-derived, or products of host-microbiota cometabolism. Furthermore, biological (BIO−) or statistical (STA-) significance analyses Sankey network visualizations were employed to clarify enriched pathways connected to significantly modified metabolites in lung tissues, emphasizing host-microbiota cometabolism and microbiota-related pathways.

### 2.10 Correlation Analysis between Microbiome and Metabolome Data Sets

We examined relationships between microbiome composition and metabolic profiles using Pearson correlation analysis. To integrate microbiome and metabolome data sets, we applied a multiomics workflow (Figure 1). We normalized microbiome taxa abundance tables with centered log-ratio (CLR) transformation and included only taxa present in ≥10 samples with a minimum read count of 2000. We also applied CLR transformation to metabolomic data and retained only features with retention times <10 min, identification scores ≥38, and KEGG pathway annotation. We identified differentially abundant features with the ALDEx2 algorithm, using an effect size ≥0.6 and Wilcoxon test *p* ≤ 0.05 for microbiome data, and an effect size ≥1.2 and *p* ≤ 0.05 for metabolite data. We calculated Pearson correlation coefficients (r) and adjusted significance values with Benjamini–Hochberg correction (adjusted *p* < 0.05). Significant correlations were visualized as clustered heatmaps to highlight association patterns.

**Figure 1:**
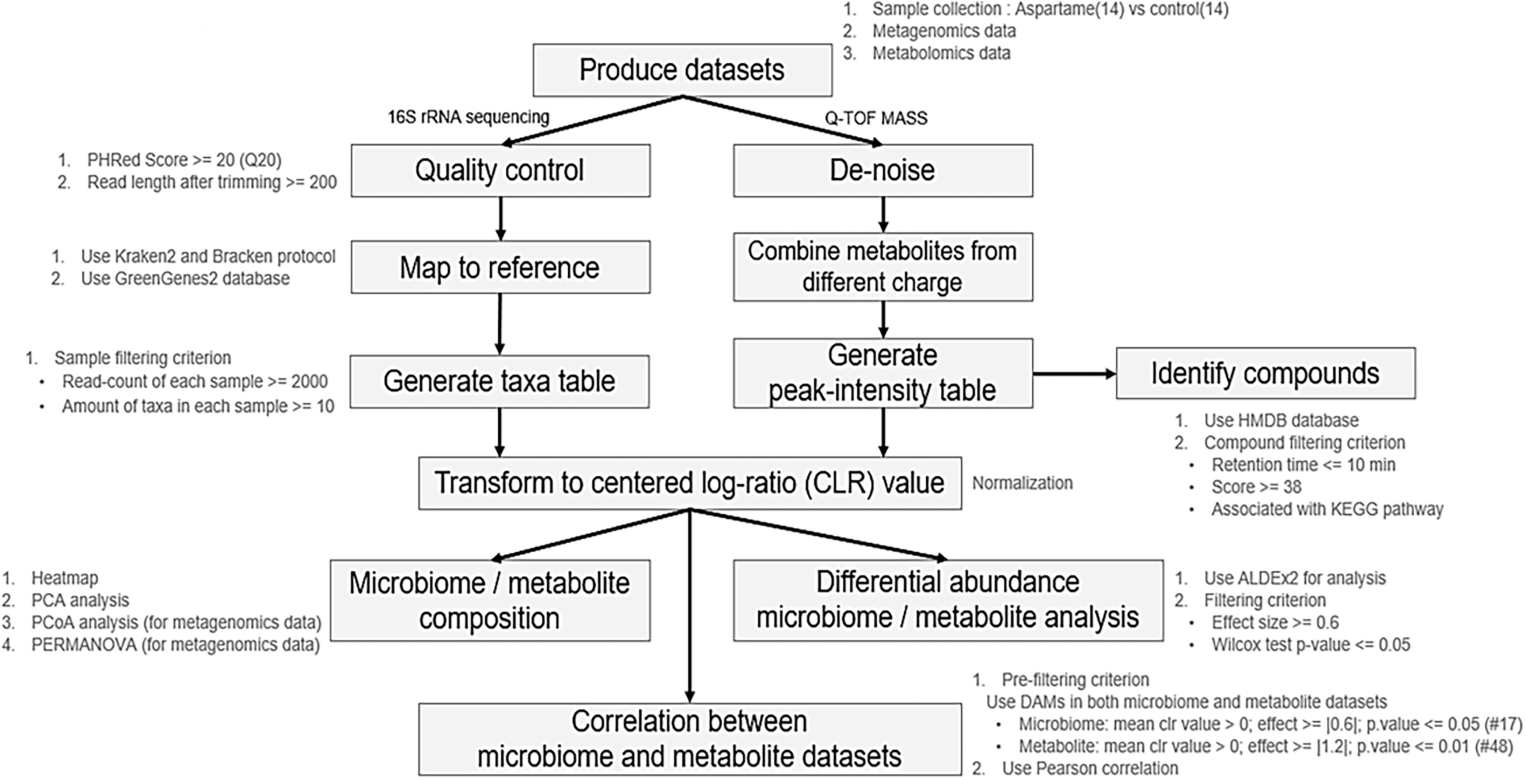
Integrated multiomics workflow for microbiome and metabolome analysis. This diagram illustrates the sequential steps involved in data acquisition, quality control, processing, and analysis for both 16S rRNA gene sequencing (microbiome) and Q-TOF mass spectrometry (metabolome) data sets. The workflow culminates in the identification of differentially abundant features and the correlation analysis between the microbiome and metabolome profiles.

### 2.11 Statistical Analysis

We expressed all data as mean ± standard deviation and considered results statistically significant at *p* < 0.05. To account for the nested study design and within-litter correlation, we analyzed all pup-level outcomes (body weight, MDA, 8-OHdG, GSH, IL-1β, ASC, and cleaved caspase-1) using linear mixed-effects models (LMMs). In these models, we included litter as a random intercept effect, and maternal aspartame exposure, pup sex, and their interaction as fixed effects. We assessed the significance of fixed effects with Type III analysis of variance using Satterthwaite’s method to estimate degrees of freedom. When models showed a “singular fit” (indicating near-zero variance of the random effect), the analysis approximated a fixed-effects model; however, we retained the LMM framework to address the hierarchical structure of the data explicitly. For significant main effects or interactions, we conducted Bonferroni-adjusted pairwise comparisons using estimated marginal means (emmeans). We reported effect sizes as partial eta squared (η_*p*_^2^). For α-diversity indices, we applied the Kruskal–Wallis test with Bonferroni correction because of their non-normal distribution. We performed all statistical tests and data visualization in R (version 4.4.3) using the lmerTest, emmeans, and effectsize packages, and in GraphPad Prism 10.1.

## 3 Result

### 3.1 Maternal Aspartame Exposure Significantly Alters Pulmonary Metabolite Profiles in Offspring on Postnatal Day 21

This study used an established animal model of maternal aspartame exposure, as described previously.^17^ To account for litter effects and ensure robust statistical analysis, all offspring analyses utilized linear mixed-effects models with litter specified as a random effect. Briefly, this model involved 8 dams (5 control, 3 aspartame-exposed) that collectively produced 56 pups (37 control, 19 aspartame-exposed) for analysis on PND 21. While no significant difference was observed in litter size, a reanalysis of offspring body weight at PND 21 using a linear mixed-effects model, which appropriately accounts for litter-specific effects, showed no significant effect of maternal aspartame exposure (F­(1, 4.286) = 2.80, *p* = 0.165, η_*p*_^2^ = 0.40), nor any significant effects of sex (F­(1, 19.712) = 0.20, *p* = 0.659, η_*p*_^2^ = 0.01) or exposure × sex interaction (F­(1, 19.712) = 0.50, *p* = 0.487, η_*p*_^2^ = 0.02).^17^

To assess metabolic differences between the groups, both PCA and PLS-DA were performed. PCA showed a clear clustering trend (PC1:62.3%, PC2:30.7%) (Figure 2A), indicating distinct metabolic profiles. PLS-DA was also conducted for exploratory visualization (Figure 2B), capturing 85.7% of the variance in a two-component model. However, cross-validation yielded negative *Q* ^2^ values and permutation testing returned a nonsignificant *p*-value (*p* = 0.368), indicating insufficient predictive validity for this supervised model. Hierarchical clustering analysis of metabolite intensities further indicated distinct clustering between the two groups (Figure 3A). Differential metabolite analysis identified 64 compounds with significant intergroup differences (fold change >2, FDR-adjusted *p*-value <0.05). After careful manual curation to exclude nonendogenous or artifactual detections, a core set of 10 biologically relevant metabolites had significant intergroup differences, as detailed in Table 1. The aspartame group exhibited elevated levels of uric acid, modified phosphatidylethanolamines, and glutathione-conjugated prostaglandins. Conversely, reductions were observed in hypoxanthine, sugar acids, phosphorylated nucleotides, diacylglycerols, and polyunsaturated fatty acids. Glycerol 3-phosphate, a phosphorylated sugar derivative, was also significantly elevated (Figure 3B, Table 1). Specifically, uric acid was significantly upregulated and hypoxanthine was significantly downregulated in the aspartame group (Figure 3C). Further statistical validation using significance analysis of metabolomics (SAM, *q*-value <0.05), followed by similar biological curation, identified a wider set of 22 significantly altered endogenous metabolites, summarized in Table S2.

**Figure 2:**
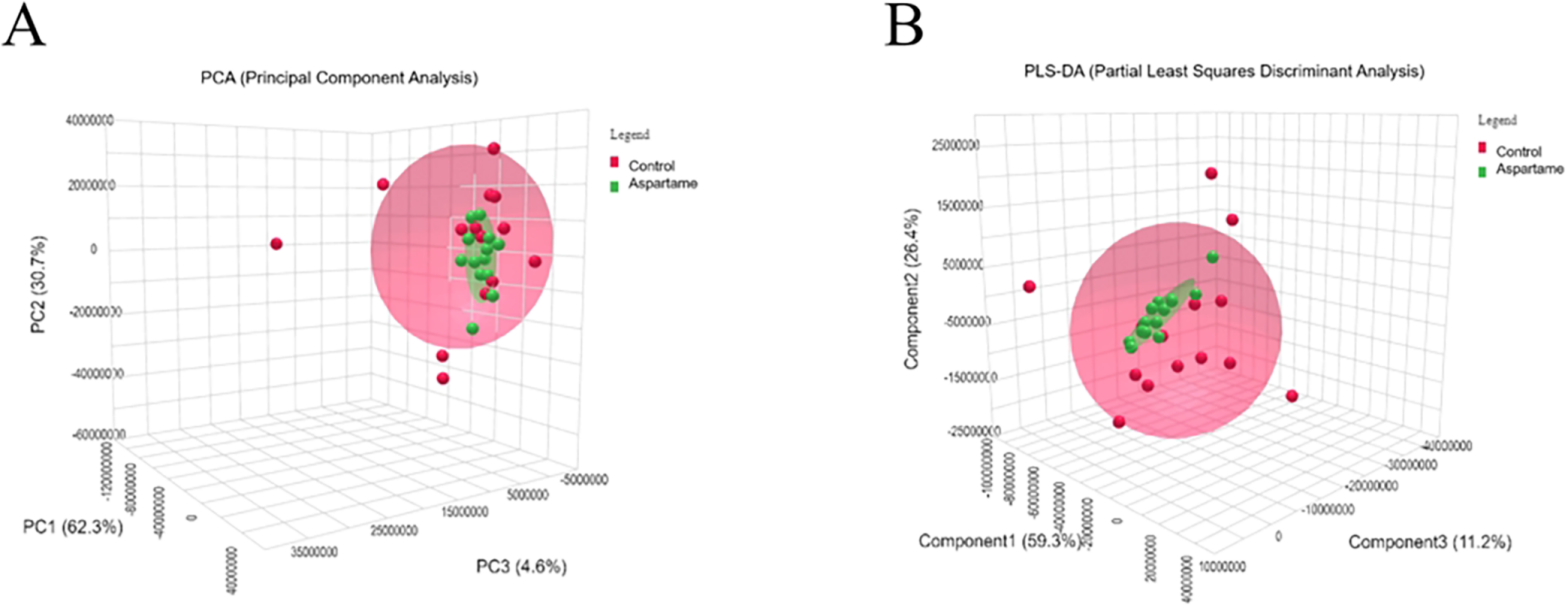
(A) Three-dimensional principal component analysis (PCA) score plots. (B) Partial least-squares discriminant analysis (PLS-DA) score plots for postnatal day 21 samples. The control group is represented by red circles, and the aspartame group is represented by green circles. The heatmap shows mean metabolite intensities in each group, with red indicating high levels and blue indicating low levels (*n* = 14).

**Figure 3:**
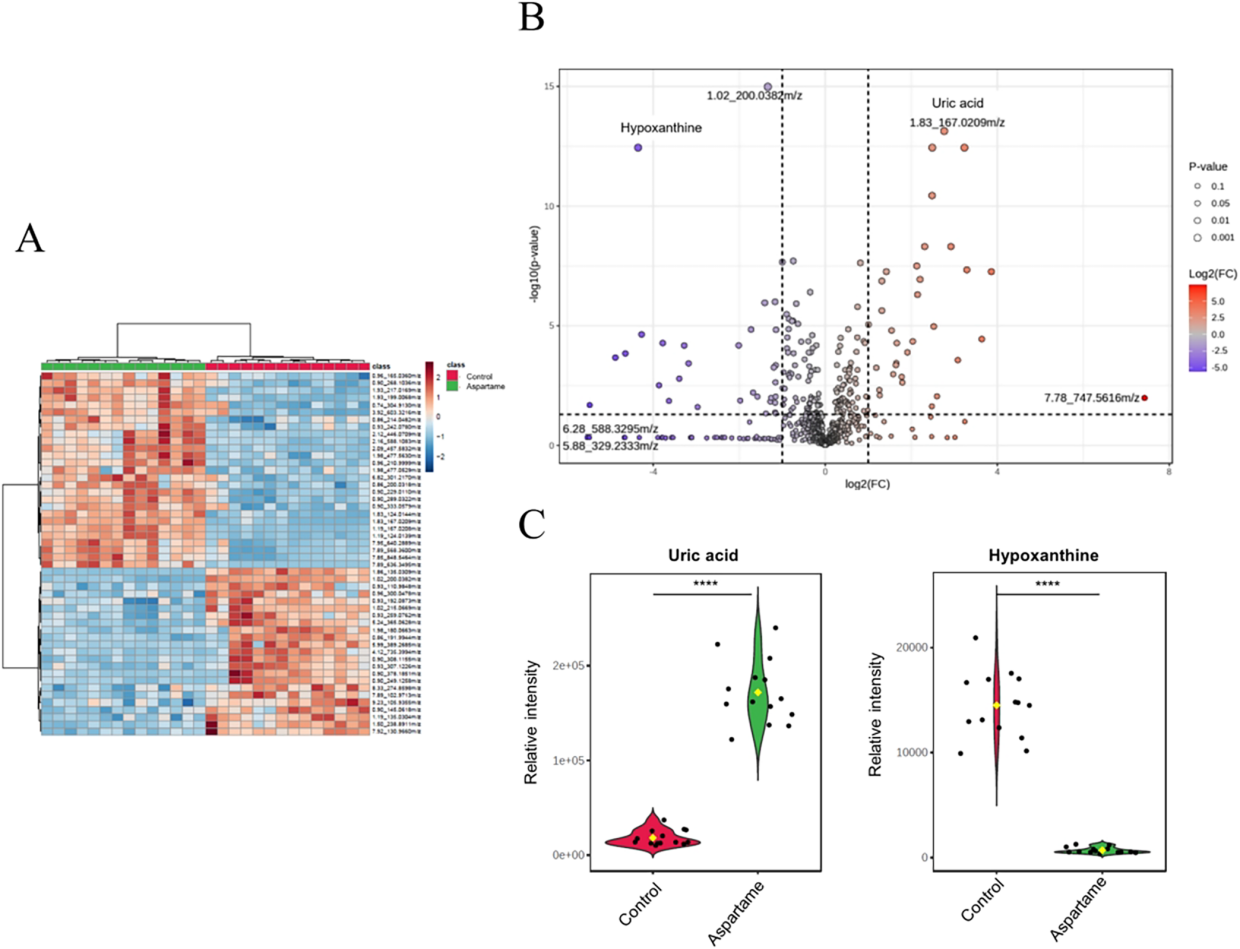
(A) Hierarchical clustering analysis of postnatal day 21 lung samples. The analysis segregated the control and aspartame groups into distinct clusters. Clustering was performed using Euclidean distance and Ward linkage. (B) Volcano plot of ultraperformance liquid chromatography–mass spectrometry (UPLC-MS/MS) data. The *y*-axis represents −log­(p) values, and the *x*-axis represents log2­(fold change). Metabolites with significant changes (fold change >2, *p* < 0.05) are highlighted in red (upregulated) and blue (downregulated), while gray dots indicate nonsignificant metabolites (*n* = 14). (C) Violin plots display the relative ion intensities of uric acid and hypoxanthine, which were significantly altered between the control and aspartame-treated groups (*n* = 14 per group). Metabolites were initially selected based on a fold change >2 and a false discovery rate (FDR)-adjusted *p*-value <0.05. Asterisks (****) indicate highly significant differences, with an FDR-adjusted *p*-value <0.0001.

**Table 1:** Ten Compounds Showed Significant Differences between the Aspartame and Control Groups in the Mice Lung (*n* = 14), as Determined by a Two-Sample *t*-Test (FDR < 0.05) and Fold Change Analysis (FC > 2)^a^

compound	FDR	identification	log 2(FC)
C1	7.27 × 10^–14^	uric acid	2.7656
C2	3.60 × 10^–13^	hypoxanthine	–4.3521
C3	1.15 × 10^–07^	PE (22:4/20:3 2OH), PE (DiMe/20:3 O), PE (DiMe/20:4 OH)	2.2056
C4	4.98 × 10^–07^	S-(9-deoxy-delta9,12-PGD2)-glutathione, S-(PGA2)-glutathione, S-(PGJ2)-glutathione, 12-Oxo-c-LTB3	2.1464
C5	9.95 × 10^–07^	2,3,4-trihydroxybutanoic acid	–1.1669
C6	0.00021337	D-myo-Inositol 1,4-bisphosphate, D-fructose 2,6-bisphosphate, fructose 1,6-bisphosphate, glyceraldehyde 3-phosphate, dihydroxyacetone phosphate, α-d-glucose 1,6-bisphosphate, D-myo-Inositol 1,3-bisphosphate, D-myo-Inositol 3,4-bisphosphate, D-tagatose 1,6-bisphosphate, D-mannose 1,6-bisphosphate, (1-Hydroxy-3-oxopropan-2-yl) dihydrogen phosphate	–4.8805
C7	0.0023005	adenosine 3′,5′-diphosphate, dGDP, ADP, [(2S,3R,4R,5R)-5-(6-aminopurin-9-yl)-3,4-dihydroxyoxolan-2-yl]methyl phosphono hydrogen phosphate	–1.1574
C8	0.0080719	glycerol 3-phosphate, β-glycerophosphoric acid, glycerophosphoric acid	1.2681
C9	0.0091006	DG(13:0/20:4+O/0:0), DG(13:0/20:5-OH/0:0), DG(13:0/20:5-OH(18)/0:0), DG(13:0/20:5-OH(12)/0:0), DG(a-13:0/20:4+O/0:0), DG(a-13:0/20:5-OH(18R)/0:0), DG(i-13:0/20:4+O/0:0), DG(i-13:0/20:5-OH(18R)/0:0)	–1.5762
C10	0.013757	arachidonic acid, Cis-8,11,14,17-eicosatetraenoic acid	–3.6345

a Compounds are numbered as C1 to C10.

### 3.2 Purine Metabolism and Pentose Phosphate Pathway Are Major Targets of Dysregulation on Postnatal Day 21

We performed a KEGG pathway enrichment analysis on all significantly altered metabolites between the control and aspartame groups, which identified 31 enriched pathways (Figure 4). Of these, four pathways were significantly impacted (FDR-adjusted *p*-value <0.05): arachidonic acid metabolism, pentose phosphate pathway, purine metabolism, and amino sugar and nucleotide sugar metabolism (Table 2). Notably, alterations in purine metabolism were characterized by increased uric acid and decreased hypoxanthine levels.

**Figure 4:**
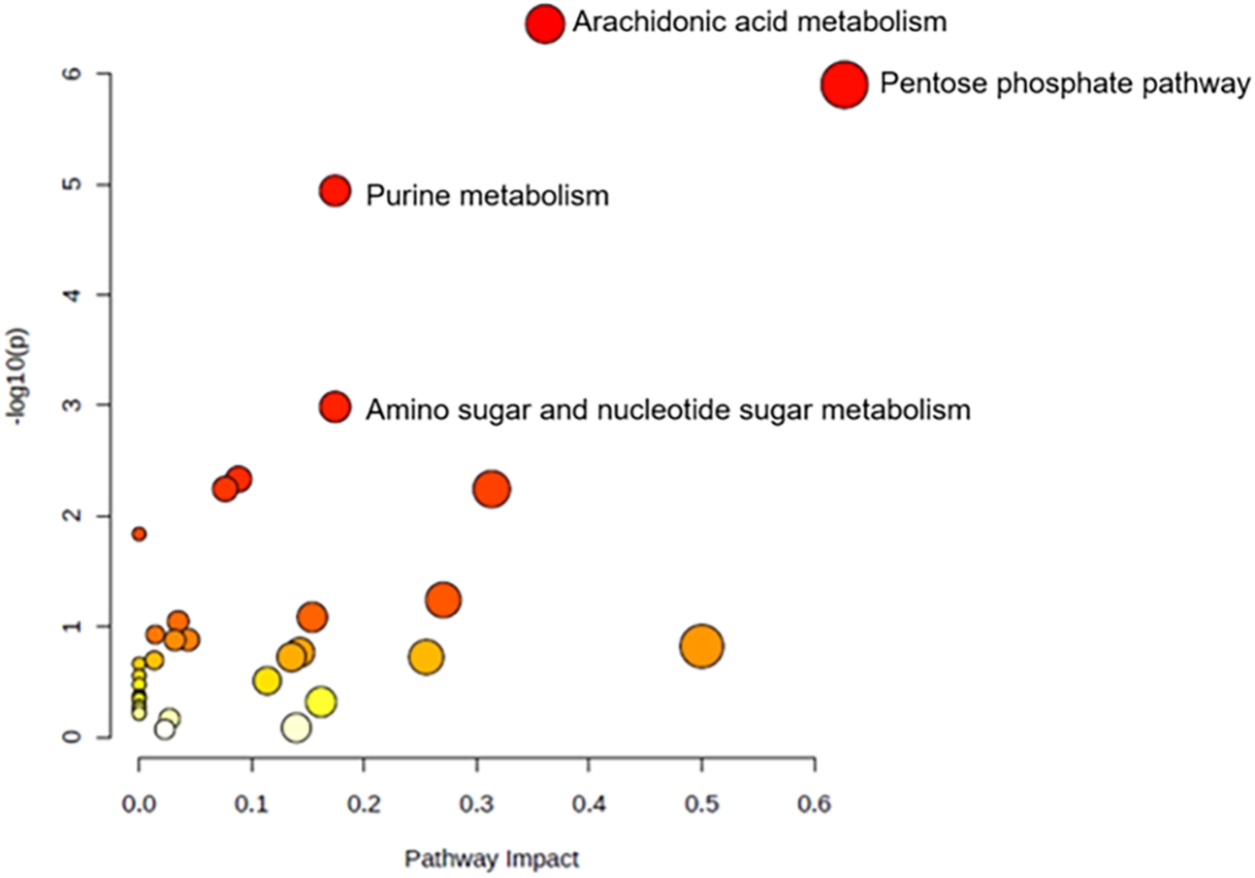
Bubble plot of metabolic pathway enrichment in offspring lungs exposed to maternal aspartame. Pathway enrichment analysis highlights metabolic pathways affected by maternal aspartame consumption in offspring lungs on postnatal day 21 (*n* = 14). Bubble color (yellow to red) indicates the *p* value (red = more significant), while bubble size represents the enrichment ratio (observed hits/expected hits).

**Table 2:** Identification of Major Differential Metabolites and Pathways in the Mice Lung Using Kyoto Encyclopedia of Genes and Genomes (*n* = 14)

pathway	match status	impact	FDR	metabolites
arachidonic acid metabolism	11/43	0.36084	2.3546 × 10^–5^	5,6-EET; 8,9-EET; 11,12-EET; 14,15-EET; Arachidonate; 16(R)-HETE; 20-HETE; 15(S)-HETE; 19(S)-HETE; 5(S)-HETE
pentose phosphate pathway	8/23	0.62684	4.3828 × 10^–5^	D-ribose 5-phosphate; α-d-ribose 1-phosphate; d-glyceraldehyde 3-phosphate; sedoheptulose 7-phosphate; D-ribulose 5-phosphate; D-fructose 1,6-bisphosphate
purine metabolism	12/71	0.17409	2.5254 × 10^–4^	xanthine; D-ribose 5-phosphate; l-glutamine; ADP; AMP; hypoxanthine; inosine; urate; dGMP; dGDP; guanosine; α-d-ribose 1-phosphate
amino sugar and nucleotide sugar metabolism	7/42	0.17414	0.018582	N-acetyl-d-glucosamine 6-phosphate; N-acetyl-α-d-glucosamine 1-phosphate; UDP-glucose; 3-deoxy-d-glycero-d-galacto-non-2-ulosonic acid; N-acetyl-α-d-galactosamine 1-phosphate; N-acetyl-d-mannosamine 6-phosphate; UDP-α-d-galactose

### 3.3 MetOrigin Analysis Suggests Potential Host-microbiota Cometabolic Interactions in Lung Metabolism

To elucidate the potential origins of significantly altered pulmonary metabolites, we conducted metabolic origin and pathway enrichment analyses using MetOrigin 2.0. The majority of enriched pathways were associated with host–microbiota cometabolism (Figure 5A). Among the significantly enriched pathways, purine metabolism and amino sugar and nucleotide sugar metabolism were selected for detailed network analysis (Table 2). Ascorbate and aldarate metabolism were additionally selected due to their significant microbial association and relevance to redox processes. Network analysis, focusing on potential cometabolic interactions within these pathways, showed significant associations between specific gut microbial genera and key metabolites (Figure 5B). In purine metabolism, correlations were observed between altered uric acid and hypoxanthine levels and specific upregulated or downregulated microbial genera. Similarly, in ascorbate and aldarate metabolism, l-gluconic acid levels exhibited significant correlations with changes in several gut microbial genera.

**Figure 5:**
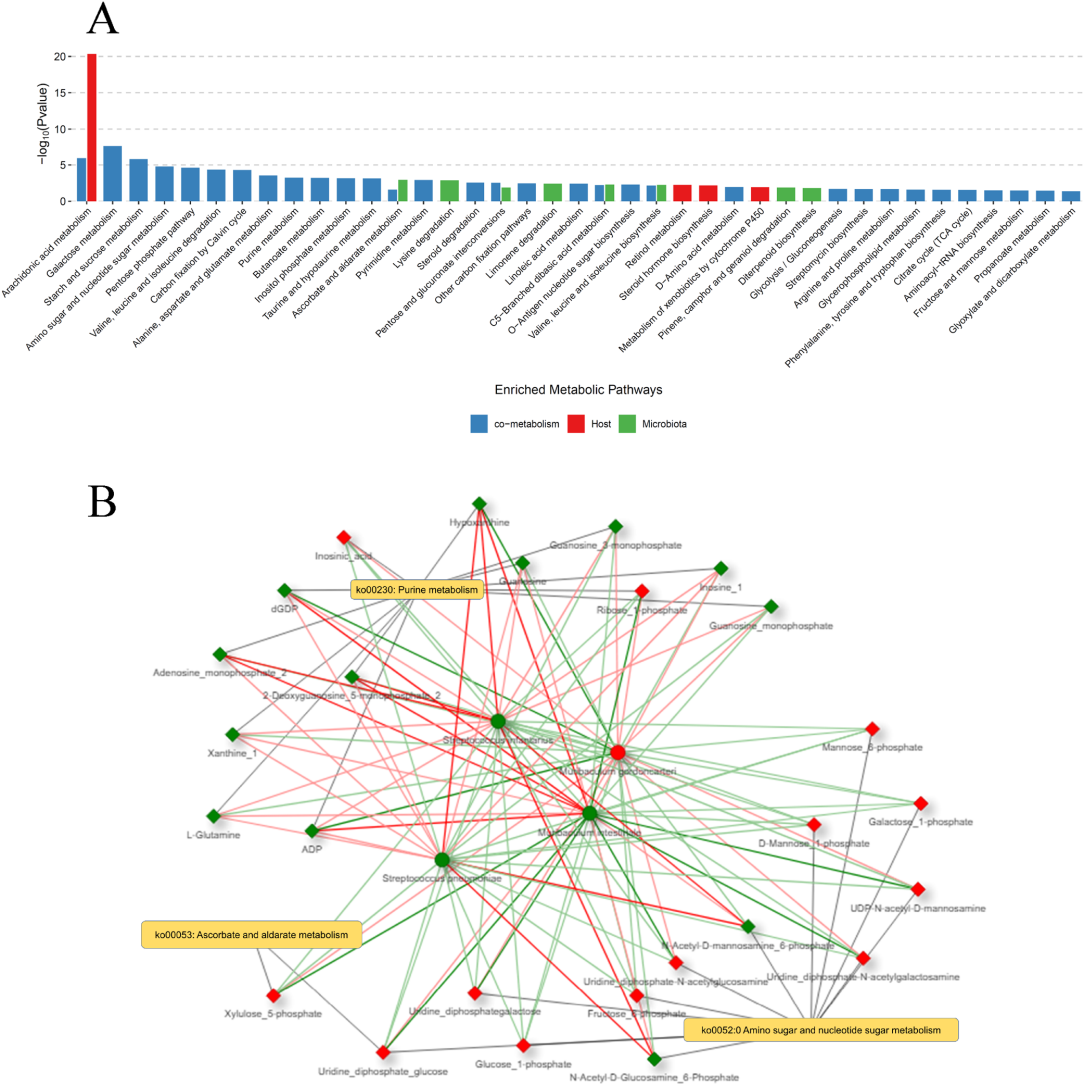
(A) Pathway enrichment analysis of differentially regulated metabolites in offspring lungs following maternal aspartame exposure. Pathways are classified as host-associated (red), microbiota-associated (green), or cometabolic (blue). Significance was assessed using the hypergeometric test, with the *Y*-axis showing log_0.05_-transformed p-values. Pathways with log_0.05_-*p*-values >1 were considered significant. (B) Network diagram of cometabolic interactions within key metabolic pathways in offspring lungs following maternal aspartame exposure. The network focuses on amino sugar and nucleotide sugar metabolism, purine metabolism, and ascorbate and aldarate metabolism—pathways identified from prior metabolomic analysis as significantly enriched and biologically associated with ten differentially abundant metabolites between aspartame and control groups (see Table 1; two-sample *t* test, FDR < 0.05; fold change >2). Diamonds represent metabolites; circles represent microbes. Node color indicates regulation and significance: dark red (upregulated, *p* < 0.05), light red (upregulated, *p* ≥ 0.05), dark green (downregulated, *p* < 0.05), light green (downregulated, *p* ≥ 0.05), and white (no change, FC = 1). Edges indicate Pearson’s correlations: dark red (significant positive), light red (nonsignificant positive), dark green (significant negative), light green (nonsignificant negative), and dark gray (no correlation).

### 3.4 Gut microbiota-Pulmonary Metabolome Associations and Network Insights in Offspring Lungs on Postnatal Day 21

Pearson correlation analysis revealed significant associations between newborn offspring gut microbial composition and lung metabolite profiles in offspring exposed to maternal aspartame (Figure 6A). Several microbial genera showed robust correlations with key metabolites implicated in purine metabolism (e.g., uric acid), gluconic acid, lipoxygenase-derived products, and intermediates of the pentose phosphate pathway. Specifically, genera such as *CAG-314*, *CAG-95*, *Enterocloster*, *Negativibacillus*, and *Parabacteroides_B_*862066 were positively correlated with uric acid, Sedoheptulose 7-phosphate, and lipoxygenase metabolites. Conversely, genera like *Acutalibacter* and *Paralachnospira* had positive associations with hypoxanthine and negative correlations with uric acid and related metabolites. Further exploration of these potential relationships was conducted through Sankey network analyses (Figure 6B,C). In purine metabolism (Figure 6B), the networks highlighted the statistically significant upregulation of uric acid (C00366) and the downregulation of its precursor, hypoxanthine (C00262). The MetOrigin analysis computationally predicted that reactions R01769 (hypoxanthine to xanthine) and R02107 (xanthine to urate), key steps in purine degradation, may be associated with these observed metabolic shifts. These metabolites had specific correlations with distinct gut microbial genera, as detailed in Figure 6B. Similarly, in ascorbate and aldarate metabolism (Figure 6C), the networks showed the upregulation of l-gluconic acid (C15930), a key metabolite in this pathway, which was also coassociated with D-tagaturonate (C00558). Both statistical (STA-) and biological (BIO−) significance analyses revealed correlations between l-gluconic acid levels and specific upregulated or downregulated microbial genera, as further illustrated in Figure 6C.

**Figure 6:**
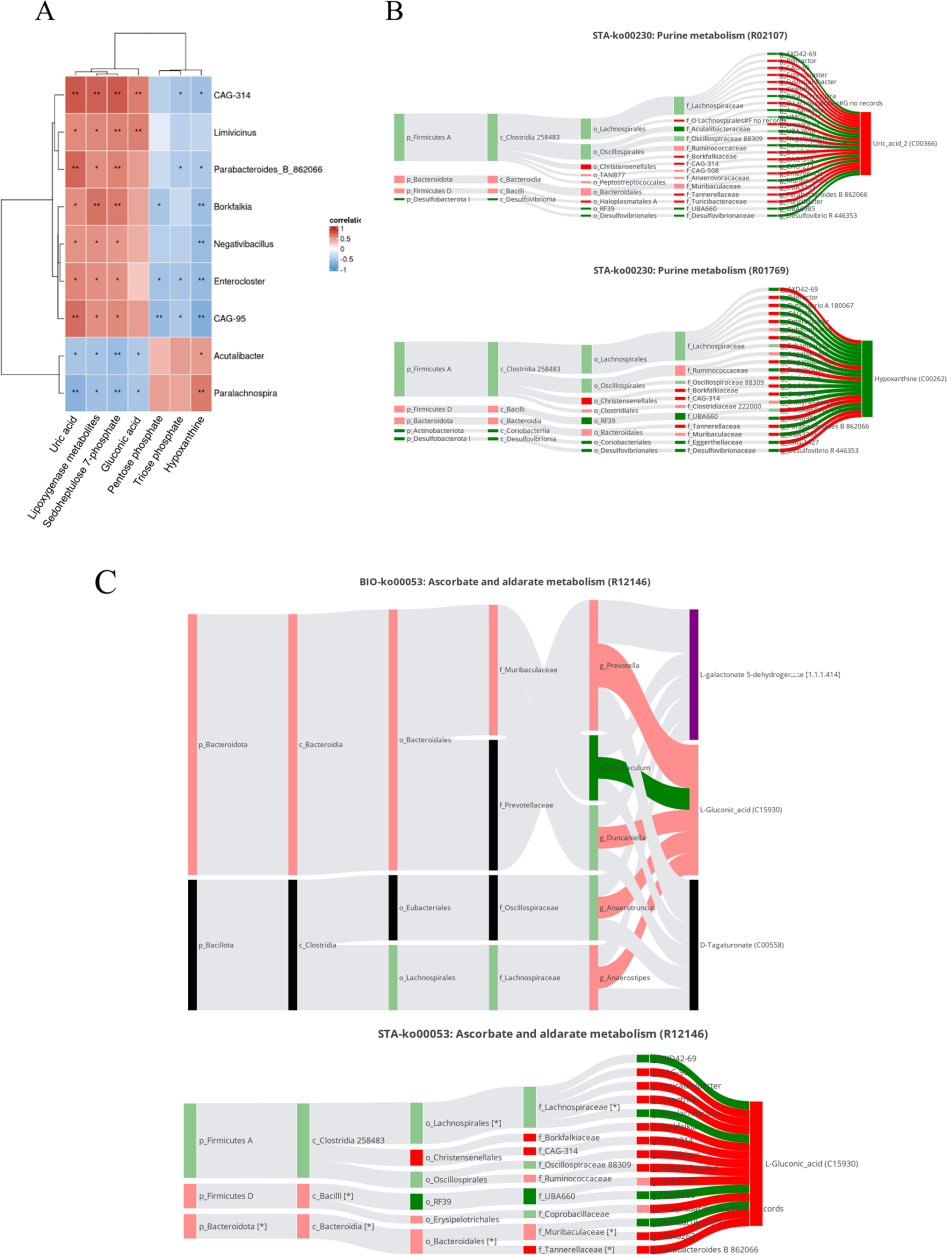
Host–microbiota interactions in offspring lungs following maternal aspartame exposure (*n* = 14 per group). (A) Pearson correlation analysis between gut microbiota (genus level) and lung metabolites. Asterisks indicate significance: *p < 0.05, **p < 0.01, ***p < 0.001. (B) Co-metabolic Sankey network within purine metabolism (STA-ko00230), illustrating the metabolic origins and flow of significantly altered metabolites. The upper panel highlights upregulation of uric acid (C00366), potentially formed via reaction R02107. The lower panel shows downregulation of hypoxanthine (C00262), potentially consumed via reaction R01769. (C) Co-metabolic Sankey network within ascorbate and aldarate metabolism (R12146), focused on l-gluconic acid (C15930) and D-tagaturonate (C00558). The upper panel (BIO-ko00053) presents a biologically significant network, showing upregulation of l-gluconic acid and its negative correlation with downregulated *Muribaculum*. The lower panel (STA-ko00053) shows a statistically significant network, including negative correlations with downregulated *Muribaculum*, *1XD42–69*, and *Paralachnospira*, and positive correlations with several upregulated genera (e.g., *CAG-95*, *Fusicatenibacter*). In MetOrigin Sankey outputs, bars represent microbes or metabolites, and connecting bands represent correlations. Red or green bars indicate up- or downregulation, respectively; darker shades denote statistical significance (*p* < 0.05), while lighter shades indicate nonsignificant changes (*p* ≥ 0.05). Bands are colored red for positive correlations and green for negative correlations, with darker shades denoting significance. Band widths are proportional to the number of microbes involved in the respective reactions.

### 3.5 Maternal Aspartame Exposure Impacts Pulmonary Oxidative Stress and Inflammasome Components in Neonatal Lungs

We further examined the direct impact of maternal aspartame exposure on lung tissue by assessing key indicators of oxidative stress and components of the inflammasome pathway. These analyses offer insights into the cellular responses that complement our previous findings on general inflammation and NF-κB activation in the same model.^17^ Oxidative stress was evaluated by measuring 8-OHdG, MDA, and GSH levels.

MDA levels were significantly elevated in offspring exposed to maternal aspartame (F­(1, 7.108) = 416.61, *p* < 0.001, η_*p*_^2^ = 0.98) (Figure 7A). No significant effects of sex (F­(1, 22.583) = 2.66, *p* = 0.117, η_*p*_^2^ = 0.11) or exposure × sex interaction (F­(1, 22.583) = 0.94, *p* = 0.343, η_*p*_^2^ = 0.04) were observed.

**Figure 7:**
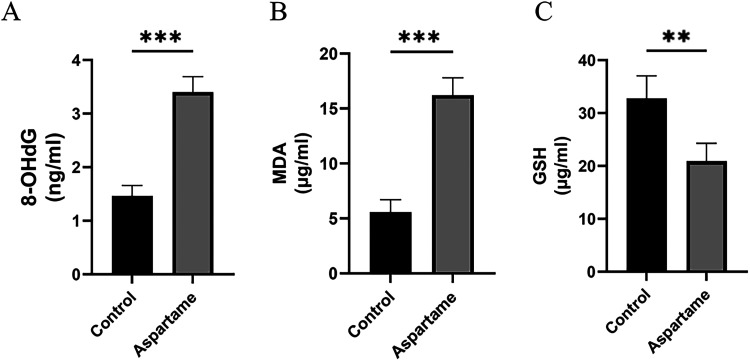
(A) Maternal aspartame exposure increases 8-hydroxy-2′-deoxyguanosine (8-OHdG) levels in offspring lungs. (B) Maternal aspartame exposure increases malondialdehyde (MDA) levels in offspring lungs. (C) Maternal aspartame exposure decreases glutathione (GSH) levels in offspring lungs. All measurements were performed using ELISA. Data are expressed as mean ± standard deviation (*n* = 14 per group). Statistical significance was determined using linear mixed-effects models for the main effect of aspartame exposure and interactions. ***p < 0.001, **p < 0.01.

Importantly, sex-specific effects were observed for oxidative DNA damage and antioxidant status. 8-OHdG levels were significantly increased by aspartame exposure (F­(1, 24) = 540.11, *p* < 0.001, η_*p*_^2^ = 0.96), with a significant exposure × sex interaction (F­(1, 24) = 8.06, *p* = 0.009, η_*p*_^2^ = 0.25), indicating a sex-dependent magnitude of response (Figure 7B, Supporting Figure S1). Post hoc comparisons showed that within the PBS group, females had significantly higher 8-OHdG than males (*t* = 2.36, df = 24, *p* = 0.027). Aspartame exposure significantly increased 8-OHdG levels in both female (*t* = 14.43, df = 24, *p* < 0.001) and male (*t* = 18.44, df = 24, *p* < 0.001) offspring when compared to their respective PBS control sexes (Supporting Figure S1).

GSH levels were significantly reduced in the aspartame group (F­(1, 4.933) = 23.51, p = 0.005, η_*p*_^2^ = 0.83), with a significant exposure × sex interaction (F­(1, 18.006) = 4.46, p = 0.049, η_*p*_^2^ = 0.20) (Figure 7C). Post hoc analysis indicated that aspartame exposure significantly reduced GSH in both female (t = −3.49, df = 7.39, *p* = 0.009) and male (*t* = −5.33, df = 7.11, *p* = 0.001) offspring compared to their respective control sexes. Within the aspartame group, a significant sex difference was observed (*t* = 2.82, df = 17.57, *p* = 0.011), with female offspring having higher GSH levels than males (Supporting Figure S1).

We then examined NLRP3 inflammasome-related proteins (Supporting Figure S2–4). ASC levels did not show a significant main effect of aspartame exposure (F­(1, 4.686) = 4.09, *p* = 0.103, η_*p*_^2^ = 0.47), sex (F­(1, 6.867) = 0.54, *p* = 0.487,, η_*p*_^2^ = 0.07), or an interaction (F­(1, 6.867) = 1.03, p = 0.343, η_*p*_^2^ = 0.13) (Figure 8A).

**Figure 8:**
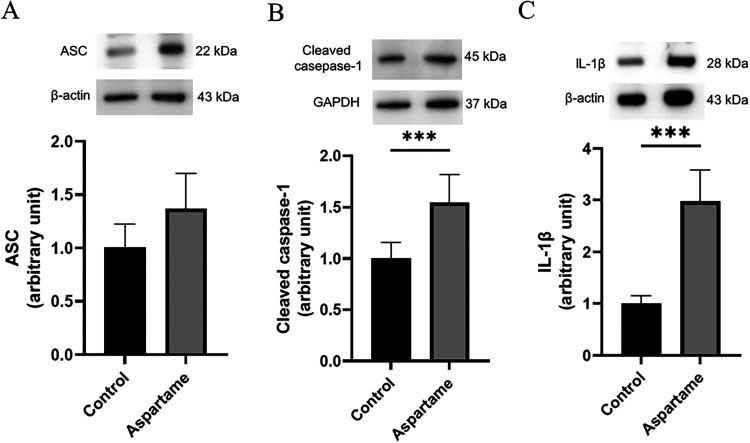
Expression of NLRP3 inflammasome-associated proteins in lung tissue of P21 offspring following maternal aspartame exposure. (A) Representative Western blot and densitometric quantification of ASC protein levels. (B) Representative Western blot and densitometric quantification of cleaved caspase-1 protein levels. (C) Representative Western blot and densitometric quantification of IL-1β protein levels. For quantitative analyses, protein levels were normalized to a loading control (β-actin or GAPDH). Quantitative analyses revealed significantly increased expression of cleaved caspase-1 and IL-1β in the aspartame-exposed group compared to controls (*n* = 8 per group). Data are presented as mean ± SD. Statistical significance was determined by linear mixed-effects models: ***p < 0.001. ASC levels did not show a statistically significant effect of aspartame exposure (*p* = 0.103).

Interestingly, sex-specific modulation of inflammasome activation was observed. Cleaved caspase-1 levels were significantly elevated following aspartame exposure ((F­(1, 12) = 49.57, *p* < 0.001, η_*p*_^2^ = 0.805), 0.81), with a significant main effect of sex (F­(1, 12) = 13.16, *p* = 0.003, η_*p*_^2^ = 0.52) (Figure 8B). The interaction approached significance (F­(1, 12) = 3.33, *p* = 0.093, η_*p*_^2^ = 0.22). Post hoc analysis indicated that female offspring had significantly higher cleaved caspase-1 levels than males (*p* = 0.0035). (Supporting Figure S3).

IL-1β levels were significantly elevated in the aspartame group (F­(1,12) = 77.33, *p* < 0.001, η_*p*_^2^ = 0.866), with no significant effects of sex (F­(1,12) = 0.61, *p* = 0.449, η_*p*_^2^ = 0.05) or interaction (F­(1,12) = 0.97, *p* = 0.345, η_*p*_^2^ = 0.07). (Figure 8C).

## 4 Discussion

This study provides new information about the association between maternal aspartame intake and disrupted pulmonary homeostasis in offspring. It specifically shows alterations in lung metabolism, oxidative stress, and NLRP3 inflammasome activation. To our knowledge, this is the first study to suggest a potential mechanistic link between maternal aspartame consumption and neonatal pulmonary redox imbalance that is associated with gut microbiota–associated metabolic signaling, thus advancing the concept of a maternal diet–microbiota–lung redox axis. PCA showed distinct clustering between aspartame-exposed and control offspring, suggesting significant metabolic remodeling in the neonatal lung. Building on earlier work,^17^ our analyses raise the hypothesis that the gut-lung axis may be involved, where correlations suggest potential associations between gut microbiota-derived metabolites and observed pulmonary changes. These results broaden the mechanistic framework of aspartame’s effects during development and emphasize the significance of assessing its impact on subclinical metabolic and inflammatory outcomes in developing organs.

Our results showed clear alterations in purine metabolism in aspartame-exposed offspring, characterized by upregulated uric acid and downregulated hypoxanthine. This metabolic shift is consistent with increased purine catabolism. Specifically, our computational MetOrigin analysis (Figure 6B) predicted potential involvement of reactions R01769 (hypoxanthine to xanthine) and R02107 (xanthine to urate), which may represent key steps in purine degradation catalyzed by xanthine oxidase. This enzyme is known for generating reactive oxygen species (ROS) during purine degradation, thereby contributing to cellular oxidative burden, an important toxicological end point. Previous studies have indicated that uric acid, recognized as a canonical DAMP, can trigger NLRP3 inflammasome activation via caspase-1–mediated IL-1β processing, particularly in pulmonary macrophages.^20,21^ These results suggest that dysregulated purine metabolism, which may be linked to increased xanthine oxidase activity and subsequent ROS production, may be associated with inflammasome activation in the developing lung following prenatal aspartame exposure. This pathway represents a potential mechanistic hypothesis linking maternal diet-associated purine catabolism with downstream oxidative stress and redox-sensitive inflammatory signaling in the lung.

Concurrently, alterations in the pentose phosphate pathway (PPP) were evident, with elevated levels of intermediates such as sedoheptulose-7-phosphate. This observation suggests impaired NADPH production and diminished antioxidant capacity,^22,23^ which is consistent with prior work showing PPP-derived NADPH’s essential role in maintaining redox balance in lung tissue.^24^ This metabolic vulnerability was supported by our observation of increased oxidative markers, including 8-OHdG (indicating DNA damage) and MDA (reflecting lipid peroxidation), alongside reduced GSH levels, suggesting diminished cellular antioxidant capacity. Importantly, while MDA and GSH showed general impacts, 8-OHdG showed sex-dependent differences, with a significant exposure × sex interaction (Figure 7B, Supporting Figure S1). The impaired PPP, when considered alongside the purine degradation changes, suggests a dual metabolic vulnerability: increased ROS production and reduced NADPH availability acting in concert to exacerbate redox imbalance and inflammasome activation. Given the redox sensitivity of NLRP3 inflammasome activation, this oxidative imbalance may contribute to inflammasome activation.^25^ Taken together, combined disruptions in purine and PPP metabolism, along with increased oxidative markers and reduced glutathione, support their connection with the observed NLRP3 inflammasome activation, as shown by significantly upregulated cleaved caspase-1 and IL-1β protein levels. Interestingly, a significant main effect of sex and an approaching interaction were observed for cleaved caspase-1, suggesting sex-specific modulation of inflammasome activation, with a greater elevation in females (Figure 8B, Supporting Figure S3).

Sex-dependent responses emerged as a key finding, with significant exposure × sex interactions observed for key biomarkers. As shown in Supporting Figure S1, control females had higher baseline 8-OHdG than males (*p* = 0.027), and both sexes showed robust increases after aspartame exposure. In the aspartame group, females maintained higher GSH levels and displayed greater increases in cleaved caspase-1 (main effect of sex, *p* = 0.003), suggesting greater susceptibility to oxidative DNA damage and more pronounced inflammasome activation compared to males.

These sex-dimorphic responses align with established biological mechanisms. Estrogen exhibits dual roles in redox regulation, both boosting antioxidant defenses via Nrf2-dependent glutathione synthesis and paradoxically promoting ROS signaling under inflammatory conditions.^26,27^ Several immune-regulatory genes on the X chromosome, including TLR7 and IRAK1, escape X-inactivation and are expressed at higher levels in females, contributing to heightened inflammasome and IL-1β responses.^28,29^ The observed sex-specific differences in GSH (interaction *p* = 0.049) and 8-OHdG (interaction *p* = 0.009) highlight the importance of considering sex as a biological variable in developmental toxicology studies of aspartame.

Disruptions in arachidonic acid metabolism were a key result (Table 2), pointing to the involvement of lipid mediators in the inflammatory phenotype. Our metabolomics analysis showed significantly altered levels of key lipoxygenase-derived products, including 12-Oxo-c-leukotriene B3, as well as numerous hydroxyeicosatetraenoic acids (HETEs) such as 5-HETE, 15-HETE, and 20-HETE (Table 1, Table S2). These specific alterations in leukotriene-related compounds and other eicosanoids indicate a pro-inflammatory lipid shift that coincides with the observed pulmonary dysregulation, and they are frequently associated with or influenced by oxidative processes.

Nucleotide sugars, as essential substrates for glycosylation, a post-translational modification known to modulate protein function and immune activation,^30,31^ also showed altered levels. These metabolic shifts therefore may contribute to inflammasome priming, a process known to involve NLRP3 based on previous studies.^32^ Taken together, these results indicate that specific metabolic pathways can lead to inflammasome activation, bringing together metabolic stress and inflammatory signals into a coordinated cellular response.

Importantly, our study indicates host–microbiota–metabolite coregulation as a potential contributing factor to this phenotype. Correlation and network analyses revealed specific and biologically plausible associations between certain gut microbial genera and key pulmonary metabolites. Consistent with our previous work showing an increase in *Negativibacillus* in aspartame-exposed offspring,^17^ this genus was positively correlated with lung uric acid, sedoheptulose 7-phosphate, and lipoxygenase metabolites. Furthermore, other genera such as *CAG-314*, *CAG-95*, *Enterocloster*, and *Parabacteroides_B_862066* were also positively correlated with lung uric acid and sedoheptulose 7-phosphate, while *Acutalibacter* and *Paralachnospira* had inverse correlations with uric acid and related metabolites. These data are consistent with the hypothesis that dietary aspartame-associated microbial signatures may be associated with host metabolic changes in distal organs, supporting the concept of a metabolite-centric gut-lung axis and microbiota-host cross-talk.^33,34^ These correlations support a potential microbiota–metabolite–host interaction framework that could be associated with maternal aspartame exposure with distal redox-sensitive immune signaling during lung development. These results of host–microbiota–metabolite coregulation prompted further investigation into downstream cellular responses, specifically oxidative stress and inflammasome activation.

These results outline a proposed mechanistic pathway wherein aspartame-induced perturbations in purine metabolism raise ROS production via xanthine oxidase, while concurrent disruptions in the pentose phosphate pathway impair NADPH-dependent antioxidant defenses, ultimately contributing to redox-sensitive inflammasome activation. This dual-hit mechanism further supports the metabolic susceptibility of the developing lung.

From a broader viewpoint, our study adds important context to existing literature on artificial sweetener safety. While many studies, especially those focusing on adult systemic metabolic outcomes under chronic exposure, report neutral or even beneficial effects of artificial sweeteners,^5^ our results reveal a distinct impact of maternal aspartame exposure specifically on neonatal lung metabolic homeostasis. This apparent difference indicates the significance of developmental susceptibility and organ-specific responses. Our multiomics approach, by studying specific pulmonary metabolic pathways and their associations with oxidative stress and inflammasome activation, uncovers subclinical metabolic and inflammatory outcomes that might be overlooked in broader systemic assessments. This work emphasizes the importance of assessing such effects induced by food components that may contribute to altered respiratory homeostasis in offspring, warranting further investigation into their long-term effects and human relevance.

Although our murine model provides useful mechanistic context, its translation to human physiology requires caution. The study design was correlative, and causal inference along the microbiota–metabolite–immune axis cannot be made from the present data. While our correlation and MetOrigin analyses suggest possible host–microbiota cometabolism, direct evidence of microbial metabolite translocation or functional impact on lung cells was not established in this study. Moreover, NLRP3 protein levels were not directly measured, so inflammasome activation cannot be definitively attributed to the canonical NLRP3 pathway. The relatively modest number of litters (n = 7) and smaller subset analyzed by Western blot may have reduced power to detect moderate effects, though large effect sizes were observed for cleaved caspase-1 and IL-1β. Sex-dependent differences were consistent across several end points, underscoring the need for larger studies to clarify sex-specific susceptibility and dose response relationships.

Future work should test causality through interventional approaches such as microbial modulation, metabolite supplementation, and inflammasome inhibition. Additional studies should also explore how microbial metabolites (e.g., short-chain fatty acids, bile acids, tryptophan catabolites) shape pulmonary immunity. Collectively, our data support a working model in which maternal aspartame exposure disrupts purine metabolism, enhancing ROS generation via xanthine oxidase, and alters the pentose phosphate pathway, reducing NADPH-dependent antioxidant defense. This dual metabolic stress may contribute to redox imbalance and inflammasome activation, providing a plausible framework for developmental lung susceptibility.

## 5 Conclusions

This multiomics study reveals the impact of maternal aspartame consumption on newborn lung metabolism and redox balance, contributing to oxidative stress and the activation of the inflammasome. The combination of metabolomics with gut microbiota-based cometabolic research suggests potential associations between the gut–lung axis and redox-sensitive metabolic pathways, especially purine metabolism and the pentose phosphate pathway. These redox-related alterations are accompanied by elevated oxidative damage markers and increased expression of inflammatory mediators in the neonatal lung.

This work advances the redox biology field by uncovering a previously unrecognized link between early life aspartame exposures and lung redox homeostasis disruption, potentially involving host–microbiota interactions. While causality remains to be fully established, our results underscore the significance of assessing redox-sensitive signaling mechanisms in developmental toxicology. Future studies using targeted metabolic flux analysis and interventional models are needed to clarify the causal relationships and therapeutic implications of these results.

## Supplementary Material



## Data Availability

Raw mass spectrometry metabolomics data have been deposited in the MetaboLights repository under the accession number MTBLS12004 (https://www.ebi.ac.uk/metabolights/MTBLS12004). Additional derived data supporting the findings are available from the corresponding author upon reasonable request.

## Notes

Methods: Aspartame dose calculation and human equivalent dose conversion. Raw ELISA standard curve data for GSH, MDA, and 8-OHdG (Tables S1.A–C). List of significantly altered metabolites between control and aspartame groups in mouse lung (Table S2). Additional figures showing oxidative stress markers, ASC, cleaved caspase-1, and IL-1β expression with uncropped blots (Figures S1–S4) (PDF)

## Abbreviations:

Th2: T-helper cell type 2
NF-κB: nuclear factor kappa-light-chain-enhancer of activated B cells
NLRP3: NLR family pyrin domain containing 3
DAMPs: danger-associated molecular patterns
ROS: reactive oxygen species
IL-1β: interleukin-1β
PND: postnatal day
MS: mass spectrometry
PCA: principal component analysis
PLS-DA: partial least-squares discriminant analysis
VIP: variable importance in projection
HMDB: human metabolome database
KEGG: kyoto encyclopedia of genes and genomes
FDR: false discovery rate
SDS-PAGE: sodium dodecyl sulfate-polyacrylamide gel electrophoresis
ASC: apoptosis-associated Speck-like protein containing a CARD
GSH: glutathione
MDA: malondialdehyde
8-OHdG: 8-hydroxy-2′-deoxyguanosine
ELISA: enzyme-linked immunosorbent assay
CLR: centered log-ratio
LMMs: linear mixed-effects models
LysoPEs: lysophosphatidylethanolamines
SAM: significance analysis of metabolomics
PPP: pentose phosphate pathway
BIO-: biological
STA-: statistical
HETEs: hydroxyeicosatetraenoic acids
PBS: phosphate-buffered saline
