# Supplementary material for: Maternal Aspartame Exposure Induces Neonatal Pulmonary Metabolic Dysregulation and Redox Imbalance: A Multiomics Investigation of Gut Microbiota-Host Interactions
Source: J Agric Food Chem. 2025 Oct 13;73(42):27012–24. doi: 10.1021/acs.jafc.5c08819 (PMC12550835; doi:10.1021/acs.jafc.5c08819)
Supplement: Supplementary file 1 [file jf5c08819_si_002.pdf]

Supporting Information for

Maternal aspartame exposure induces neonatal pulmonary metabolic dysregulation and redox imbalance: a multi-omics investigation of gut microbiota-host interactions

Sheng-Yuan Ho[1,2,3], Cheng-Yang Lee[4], Hsiu-Chu Chou[5], Liang-Ti Huang[6,7,*], Chung-Ming Chen[7,8,9,*]

[1]*Graduate Institute of Clinical Medicine, College of Medicine, Taipei Medical University, Taipei 11031, Taiwan*

[2]*Department of Pediatrics, Tri-Service General Hospital, National Defense Medical University, Taipei 11490, Taiwan*

[3]*Department of Pediatrics, School of Medicine, College of Medicine, National Defense Medical University, Taipei 11031, Taiwan*

[4]*Bioinformatics Center, Office of Data Science, Taipei Medical University, Taipei 11031, Taiwan*

[5]*Department of Anatomy and Cell Biology, School of Medicine, College of Medicine, Taipei Medical University, Taipei 11031, Taiwan*

[6]*Department of Pediatrics, Wan Fang Hospital, Taipei Medical University, Taipei 11696, Taiwan*

[7]*Department of Pediatrics, School of Medicine, College of Medicine, Taipei Medical University, Taipei 11031, Taiwan*

[8]*Department of Pediatrics, Taipei Medical University Hospital, Taipei 11031, Taiwan*

[9]*TMU Research Center for Digestive Medicine, Taipei Medical University, Taipei 11031, Taiwan*

*Corresponding authors. *E-mail addresses:* cmchen@tmu.edu.tw (CM. Chen), a9309@tmu.edu.tw (LT. Huang).

Table S1-1. Raw optical density (OD) values and regression statistics for GSH ELISA standard curves

Concentration (µg/mL)	Replicates (OD 450 nm)	Mean OD
200	2.372, 2.673	2.523
100	1.553, 1.432	1.493
50	0.819, 0.937	0.879
25	0.453, 0.542	0.498
12.5	0.322, 0.299	0.311
6.25	0.264, 0.271	0.268
3.13	0.107, 0.065	0.086
0	0.042, 0.045	0.044

Linear regression: $y = 0.0121x + 0.1796$, $R^2 = 0.9905$

Table S1-2. Raw optical density (OD) values and regression statistics for MDA ELISA standard curves

Concentration (µg/mL)	Replicates (OD 450 nm)	Mean OD
100	2.637, 2.584	2.611
50	1.464, 1.563	1.514
25	0.872, 0.833	0.853
12.5	0.573, 0.673	0.623
6.25	0.363, 0.332	0.348
3.13	0.263, 0.282	0.273
0	0.064, 0.053	0.059

Linear regression: $y = 0.0247x + 0.2029$, $R^2 = 0.9908$

Table S1-3. Raw optical density (OD) values and regression statistics for 8-OHdG ELISA standard curves

Concentration (ng/mL)	Replicates (OD 450 nm)	Mean OD
20	2.445, 2.378	2.412
10	1.311, 1.298	1.305
5	0.687, 0.691	0.689
2.5	0.334, 0.355	0.345
1.25	0.189, 0.179	0.184
0.625	0.146, 0.178	0.162
0	0.056, 0.044	0.050

Linear regression: $y = 0.1186x + 0.068$, $R^2 = 0.9986$

Table S2. Significantly altered database-annotated metabolites between the aspartame and control groups in the mice lung (n = 14), as determined by SAM (significance analysis of metabolomics; q-value < 0.05).

Compound	d.value	q-value	Identification
C1	5.1118	<0.001	D-Ribulose 5-phosphate; Xylulose 5-phosphate; Ribose 1-phosphate; D-Ribose 5-phosphate; D-Ribose 1-phosphate; Phosphoribose
C2	-9.5338	<0.001	Hypoxanthine; 2-Hydroxypurine; 9H-Purine-9-ol
C3	6.8787	<0.001	S-(9-deoxy-delta9,12-PGD2)-glutathione; S-(PGA2)-glutathione; S-(PGJ2)-glutathione; 12-Oxo-c-LTB3
C4	7.7752	<0.001	PE(22:4(7Z,10Z,13Z,16Z)/20:3(8Z,11Z,14Z)-2OH(5,6)); PE(20:3(8Z,11Z,14Z)-2OH(5,6)/22:4(7Z,10Z,13Z,16Z)); PE(DiMe(11,5)/20:3(5Z,8Z,11Z)-O(14R,15S)); PE(20:3(5Z,8Z,11Z)-O(14R,15S)/DiMe(11,5)); PE(DiMe(11,5)/20:3(5Z,8Z,14Z)-O(11S,12R)); PE(20:3(5Z,8Z,14Z)-O(11S,12R)/DiMe(11,5)); PE(DiMe(11,5)/20:3(5Z,11Z,14Z)-O(8,9)); PE(20:3(5Z,11Z,14Z)-O(8,9)/DiMe(11,5)); PE(DiMe(11,5)/20:3(8Z,11Z,14Z)-O(5,6)); PE(20:3(8Z,11Z,14Z)-O(5,6)/DiMe(11,5)); PE(DiMe(11,5)/20:4(5Z,8Z,11Z,14Z)-OH(20)); PE(20:4(5Z,8Z,11Z,14Z)-OH(20)/DiMe(11,5)); PE(DiMe(11,5)/20:4(6E,8Z,11Z,14Z)-OH(5S)); PE(20:4(6E,8Z,11Z,14Z)-OH(5S)/DiMe(11,5)); PE(DiMe(11,5)/20:4(5Z,8Z,11Z,14Z)-OH(19S)); PE(20:4(5Z,8Z,11Z,14Z)-OH(19S)/DiMe(11,5)); PE(DiMe(11,5)/20:4(5Z,8Z,11Z,14Z)-OH(18R)); PE(20:4(5Z,8Z,11Z,14Z)-OH(18R)/DiMe(11,5)); PE(DiMe(11,5)/20:4(5Z,8Z,11Z,14Z)-OH(17)); PE(20:4(5Z,8Z,11Z,14Z)-OH(17)/DiMe(11,5)); PE(DiMe(11,5)/20:4(5Z,8Z,11Z,14Z)-OH(16R)); PE(20:4(5Z,8Z,11Z,14Z)-OH(16R)/DiMe(11,5)); PE(DiMe(11,5)/20:4(5Z,8Z,11Z,13E)-OH(15S)); PE(20:4(5Z,8Z,11Z,13E)-OH(15S)/DiMe(11,5)); PE(DiMe(11,5)/20:4(5Z,8Z,10E,14Z)-OH(12S)); PE(20:4(5Z,8Z,10E,14Z)-OH(12S)/DiMe(11,5)); PE(DiMe(11,5)/20:4(5E,8Z,12Z,14Z)-OH(11R)); PE(20:4(5E,8Z,12Z,14Z)-OH(11R)/DiMe(11,5)); PE(DiMe(11,5)/20:4(5Z,7E,11Z,14Z)-OH(9)); PE(20:4(5Z,7E,11Z,14Z)-

			OH(9)/DiMe(11,5))
C5	15.696	<0.001	Uric acid
C6	6.6748	<0.001	D-Sedoheptulose 7-phosphate; Sedoheptulose 7-phosphate
C7	-4.227	<0.001	D-myo-Inositol 1,4-bisphosphate; D-Fructose 2,6-bisphosphate; Fructose 1,6-bisphosphate; Glyceraldehyde 3-phosphate; Dihydroxyacetone phosphate; alpha-D-Glucose 1,6-bisphosphate; D-myo-Inositol 1,3-bisphosphate; D-myo-Inositol 3,4-bisphosphate; D-Tagatose 1,6-bisphosphate; D-Mannose 1,6-bisphosphate; (1-Hydroxy-3-oxopropan-2-yl) dihydrogen phosphate; 1,6-Di-O-phosphono-D-fructose; Fructose-1,6-diphosphate; Di-phosphofructose; Diphosphoglucose; [(2R,3R)-2,3,5-Trihydroxy-4-oxo-6-phosphonooxyhexyl] dihydrogen phosphate
C8	3.9966	<0.001	5,6-Epoxy-8,11,14-eicosatrienoic acid; 8,9-Epoxyeicosatrienoic acid; 14R,15S-EpETrE; 15-HETE; 14,15-Epoxy-5,8,11-eicosatrienoic acid; 11,12-Epoxyeicosatrienoic acid; 8-HETE; 16(R)-HETE; 11(R)-HETE; 20-Hydroxyeicosatetraenoic acid; 12-HETE; 18-Hydroxyarachidonic acid; 9-HETE; 5-HETE; 19(S)-HETE; 10-HETE; 13-HETE; 17-HETE; 12 Hydroxy arachidonic acid; 12S-hydroxy-5E,8Z,10Z,14Z-eicosatetraenoic acid; 15R-hydroxy-5Z,8Z,11Z,13E-eicosatetraenoic acid; 18-Hydroxy-5Z,8Z,11Z,14Z-eicosatetraenoic acid; 7-HETE; 8-hydroxy-5Z,9E,11Z,14Z-eicosatetraenoic acid; (12S)-12-Hydroxyicosa-2,4,6,8-tetraenoic acid; (14R,15S)-14,15-Epoxy-5,8,11-icosatrienoic acid; (5E,8E,11E,14E)-19-Hydroxyicosa-5,8,11,14-tetraenoic acid; 11-Hydroxy-5Z,8Z,11E,14Z-eicosatetraenoic acid; 11,12-Epoxyeicosantrienoic acid; 11R,12S-EpETrE; 12(S)-Hydroxy-5,8,10,14-eicosatetraenoic acid; 15-Hydroxy-5,8,11,13-eicosatetraenoic acid; Eicosatetraenoic acid, 15-hydroxy-; 18-Hydroxyeicosatetraenoic acid; 19-Hydroxyeicosatetraenoic acid; 20-Hydroxyarachlidonic acid; 20-Hydroxyeicosatetraenic acid; 8-Hydroxy-5E,9Z,11Z,14Z-eicosatetraenoic acid; arachidonic acid hydroperoxide; Epoxyeicosatrienoic acid; 5-Oxoicosa-3,6,8-trienoic acid
C9	3.9277	<0.001	Cytidine monophosphate; Cytidine 2'-phosphate; Cytidine 3'-monophosphate

C10	3.7784	<0.001	Cytidine
C11	-3.706	<0.001	Xanthine
C12	-3.7168	<0.001	Guanosine; 8-OHdG
C13	-3.7139	<0.001	AMP; dGMP; 3'-AMP; Adenosine 2'-phosphate; 2-hydroxy-dAMP
C14	-3.4727	<0.001	DG(13:0/20:4(5Z,8Z,11Z,13E)+=O(15)/0:0); DG(20:4(5Z,8Z,11Z,13E)+=O(15)/13:0/0:0); DG(13:0/0:0/20:4(5Z,8Z,11Z,13E)+=O(15)); DG(20:4(5Z,8Z,11Z,13E)+=O(15)/0:0/13:0); DG(13:0/20:5(5Z,8Z,11Z,14Z,16E)-OH(18R)/0:0); DG(20:5(5Z,8Z,11Z,14Z,16E)-OH(18R)/13:0/0:0); DG(13:0/0:0/20:5(5Z,8Z,11Z,14Z,16E)-OH(18R)); DG(20:5(5Z,8Z,11Z,14Z,16E)-OH(18R)/0:0/13:0); DG(13:0/20:5(5Z,8Z,11Z,14Z,16E)-OH(18)/0:0); DG(20:5(5Z,8Z,11Z,14Z,16E)-OH(18)/13:0/0:0); DG(13:0/0:0/20:5(5Z,8Z,11Z,14Z,16E)-OH(18)); DG(20:5(5Z,8Z,11Z,14Z,16E)-OH(18)/0:0/13:0); DG(13:0/20:5(5Z,8Z,10E,14Z,17Z)-OH(12)/0:0); DG(20:5(5Z,8Z,10E,14Z,17Z)-OH(12)/13:0/0:0); DG(13:0/0:0/20:5(5Z,8Z,10E,14Z,17Z)-OH(12)); DG(20:5(5Z,8Z,10E,14Z,17Z)-OH(12)/0:0/13:0); DG(a-13:0/20:4(5Z,8Z,11Z,13E)+=O(15)/0:0); DG(20:4(5Z,8Z,11Z,13E)+=O(15)/a-13:0/0:0); DG(a-13:0/0:0/20:4(5Z,8Z,11Z,13E)+=O(15)); DG(20:4(5Z,8Z,11Z,13E)+=O(15)/0:0/a-13:0); DG(a-13:0/20:5(5Z,8Z,11Z,14Z,16E)-OH(18R)/0:0); DG(20:5(5Z,8Z,11Z,14Z,16E)-OH(18R)/a-13:0/0:0); DG(a-13:0/0:0/20:5(5Z,8Z,11Z,14Z,16E)-OH(18R)); DG(20:5(5Z,8Z,11Z,14Z,16E)-OH(18R)/0:0/a-13:0); DG(a-13:0/20:5(5Z,8Z,11Z,14Z,16E)-OH(18)/0:0); DG(20:5(5Z,8Z,11Z,14Z,16E)-OH(18)/a-13:0/0:0); DG(a-13:0/0:0/20:5(5Z,8Z,11Z,14Z,16E)-OH(18)); DG(20:5(5Z,8Z,11Z,14Z,16E)-OH(18)/0:0/a-13:0); DG(a-13:0/20:5(5Z,8Z,10E,14Z,17Z)-OH(12)/0:0); DG(20:5(5Z,8Z,10E,14Z,17Z)-OH(12)/a-13:0/0:0); DG(a-13:0/0:0/20:5(5Z,8Z,10E,14Z,17Z)-OH(12)); DG(20:5(5Z,8Z,10E,14Z,17Z)-OH(12)/0:0/a-13:0); DG(i-13:0/20:4(5Z,8Z,11Z,13E)+=O(15)/0:0); DG(20:4(5Z,8Z,11Z,13E)+=O(15)/i-13:0/0:0); DG(i-13:0/0:0/20:4(5Z,8Z,11Z,13E)+=O(15)); DG(20:4(5Z,8Z,11Z,13E)+=O(15)/0:0/i-

			13:0); DG(i-13:0/20:5(5Z,8Z,11Z,14Z,16E)-OH(18R)/0:0); DG(20:5(5Z,8Z,11Z,14Z,16E)-OH(18R)/i-13:0/0:0); DG(i-13:0/0:0/20:5(5Z,8Z,11Z,14Z,16E)-OH(18R)); DG(20:5(5Z,8Z,11Z,14Z,16E)-OH(18R)/0:0/i-13:0); DG(i-13:0/20:5(5Z,8Z,11Z,14Z,16E)-OH(18)/0:0); DG(20:5(5Z,8Z,11Z,14Z,16E)-OH(18)/i-13:0/0:0); DG(i-13:0/0:0/20:5(5Z,8Z,11Z,14Z,16E)-OH(18)); DG(20:5(5Z,8Z,11Z,14Z,16E)-OH(18)/0:0/i-13:0); DG(i-13:0/20:5(5Z,8Z,10E,14Z,17Z)-OH(12)/0:0); DG(20:5(5Z,8Z,10E,14Z,17Z)-OH(12)/i-13:0/0:0); DG(i-13:0/0:0/20:5(5Z,8Z,10E,14Z,17Z)-OH(12)); DG(20:5(5Z,8Z,10E,14Z,17Z)-OH(12)/0:0/i-13:0)
C15	-3.1387	<0.001	L-Glutamine; Ureidoisobutyric acid; D-Glutamine; Alanylglycine; Cyclic Urea; Glycyl-D-Alanine; Glycylsarcosine; D-Alanyl glycine; 3-ureido-isobutyrate
C16	3.1596	<0.001	UDP-glucose; UDP-galactose
C17	3.1621	<0.001	6-Keto-prostaglandin F1a; Thromboxane B2; 20-Hydroxy-PGF2a; 10,11-dihydro-20-dihydroxy-LTB4; Prostaglandin G1; 19-hydroxyprostaglandin H1(1-); 19-Hydroxyprostaglandin E1; 19-Hydroxyprostaglandin F; 6,15-Diketo-13,14-dihydro-PGF1alpha
C18	3.0209	<0.01	Glycerylphosphorylethanolamine
C19	-3.0055	<0.01	Arachidonic acid; Cis-8,11,14,17-Eicosatetraenoic acid; 5,8,11,14-Icosatetraenoic Acid; Eicosatetraenoic acid
C20	2.9901	<0.01	Glycerophosphoglycerol
C21	-2.8808	<0.01	Deuteroporphyrin IX; Deuteroporphyrin
C22	-2.8754	<0.01	Inosine

This table lists metabolites with significantly different abundance (q-value < 0.05, SAM) between the aspartame and control groups in the mice lung, ranked by absolute SAM statistic (d. value). log2 fold change (FC) was calculated from raw data. Compounds are numbered as C1 to C22.

Figure S1.A

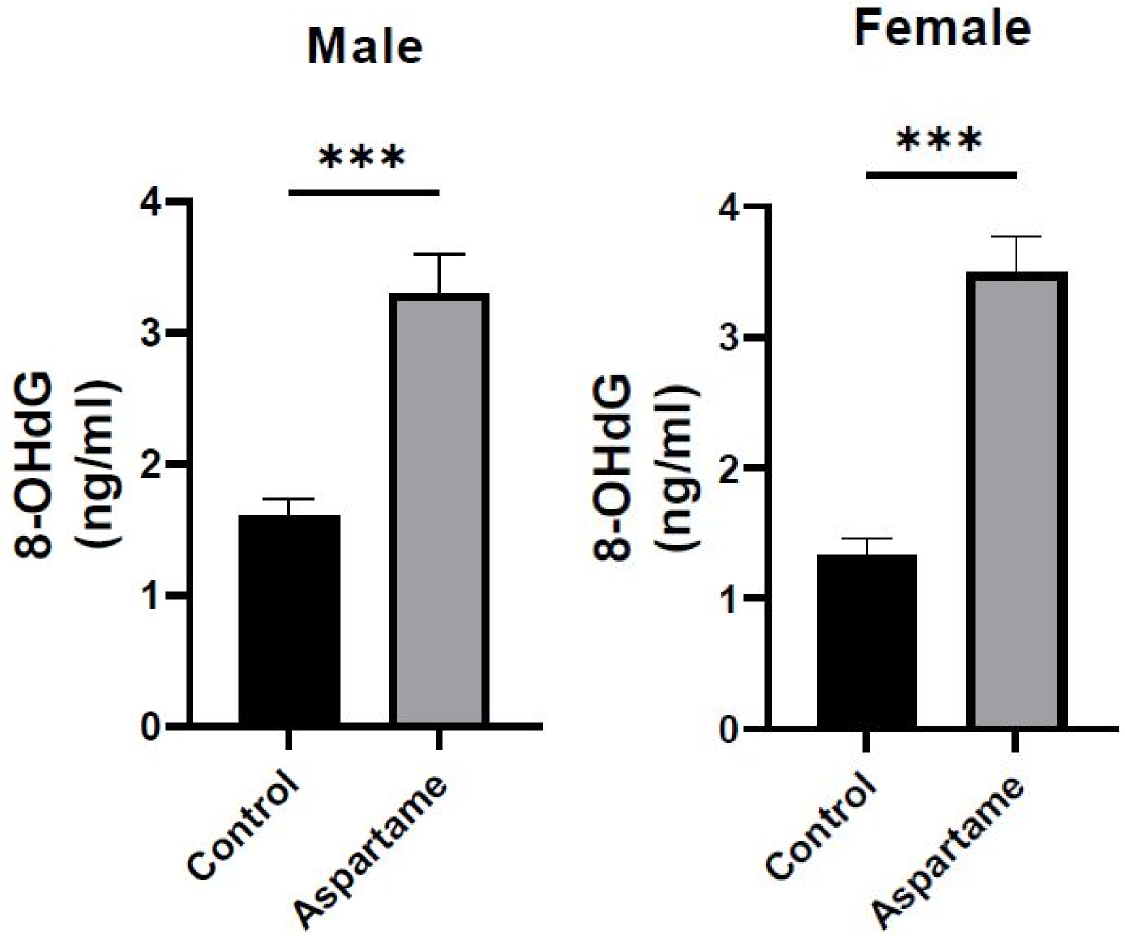

Figure S1.B

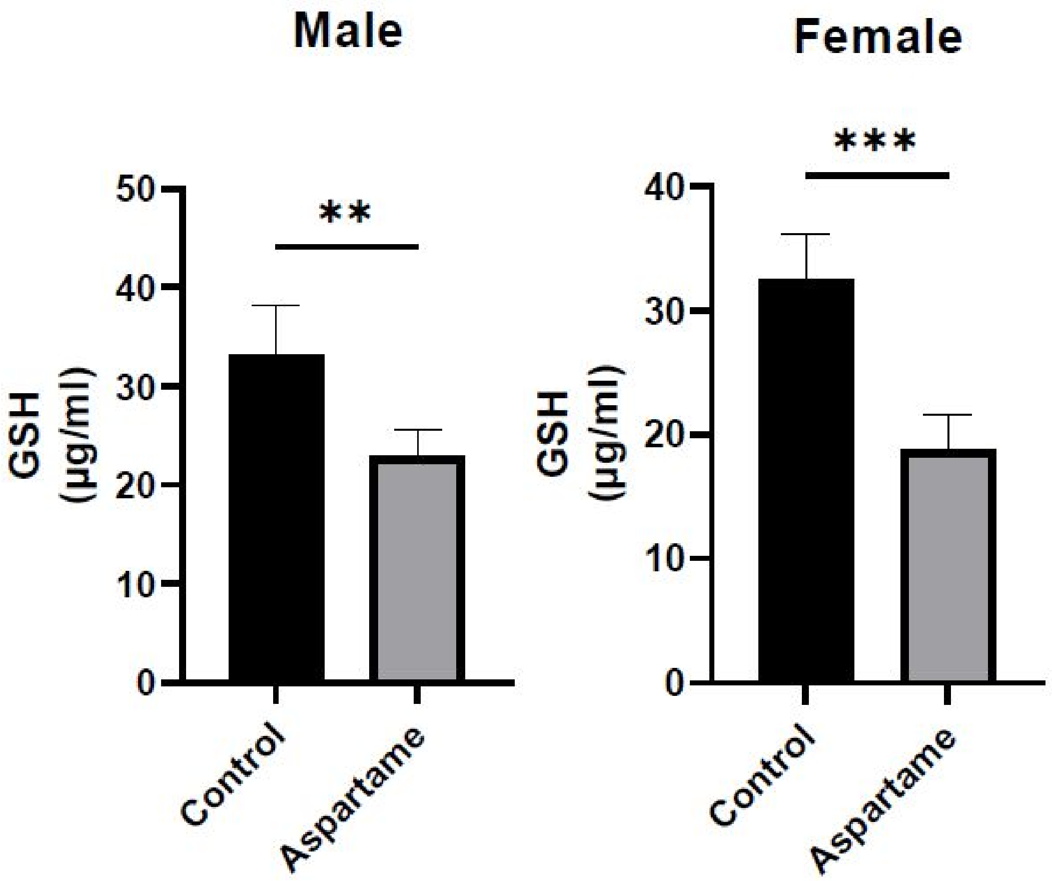

Figure S2.

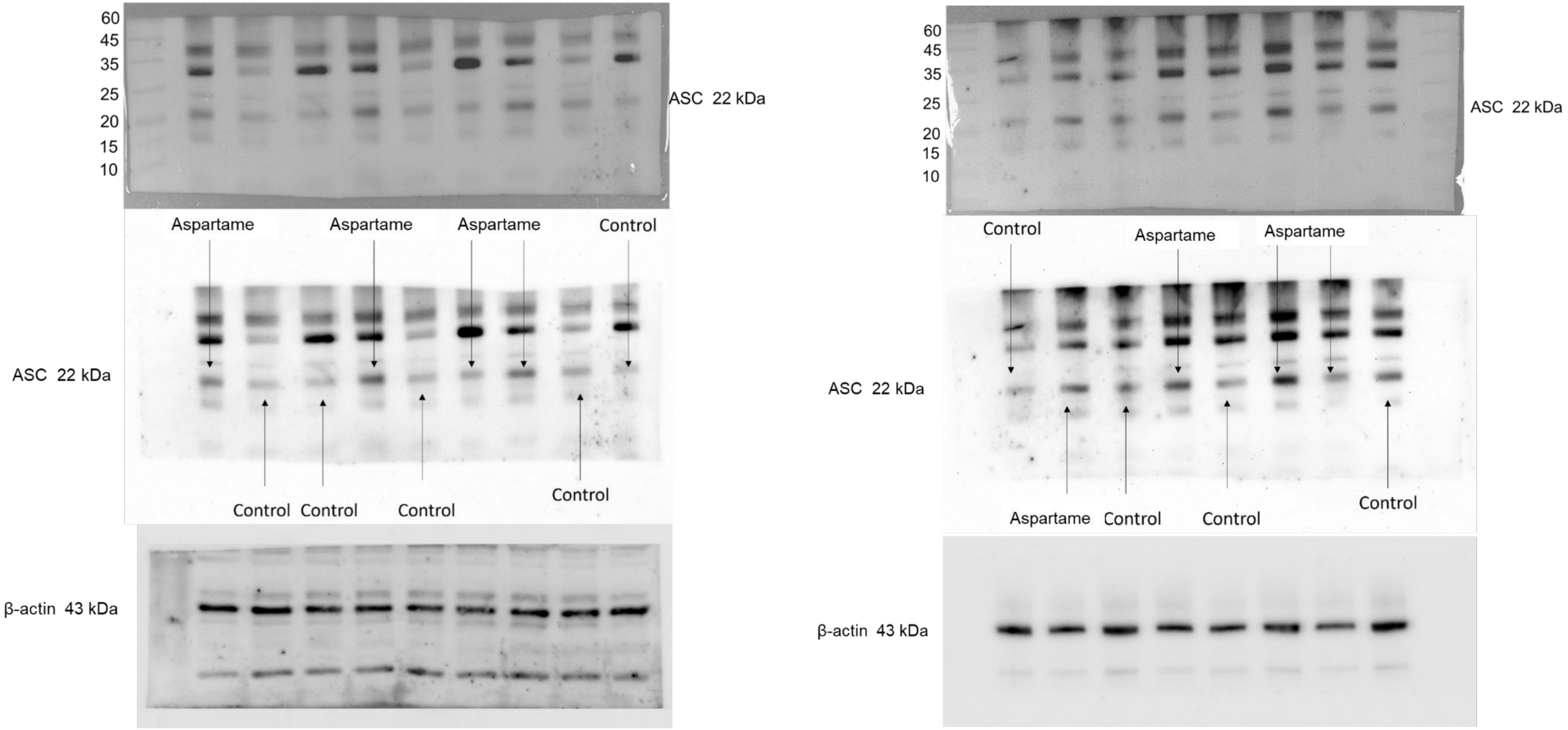
60
45
35
25
20
15
10
ASC 22 kDa
Aspartame
Aspartame
Aspartame
Control
ASC 22 kDa
Control
Control
Control
Control
β-actin 43 kDa
60
45
35
25
20
15
10
ASC 22 kDa
Control
Aspartame
Aspartame
ASC 22 kDa
Aspartame
Control
Control
Control
β-actin 43 kDa

Figure S3.

A.

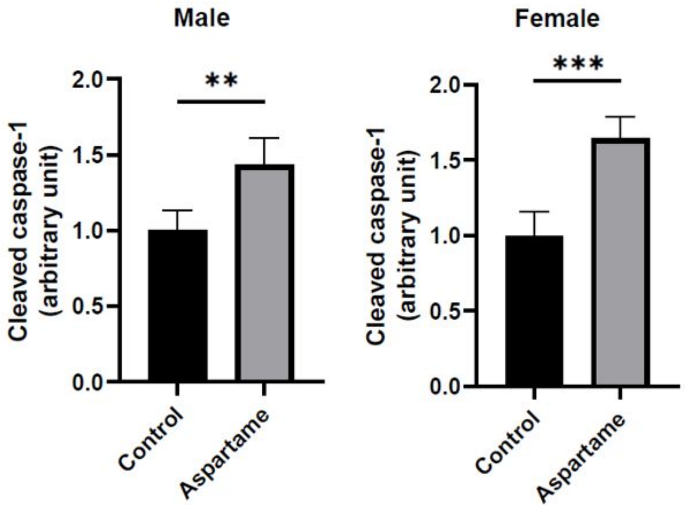

B.

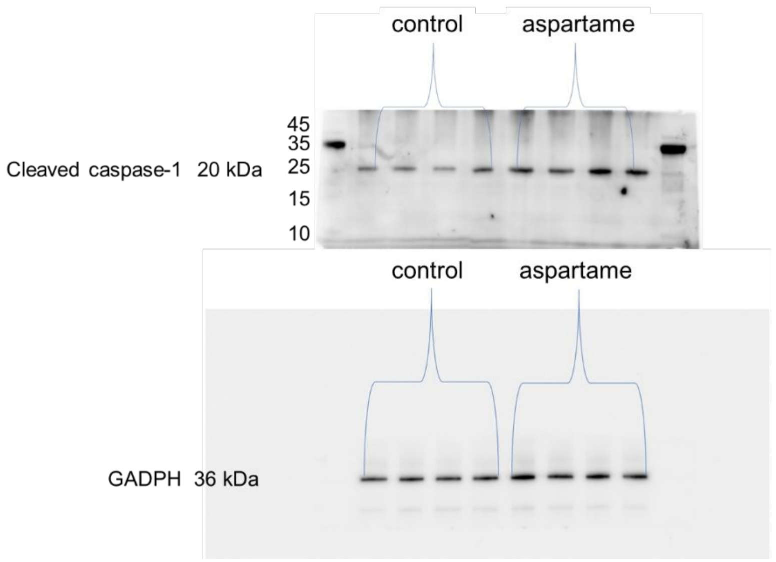

C.

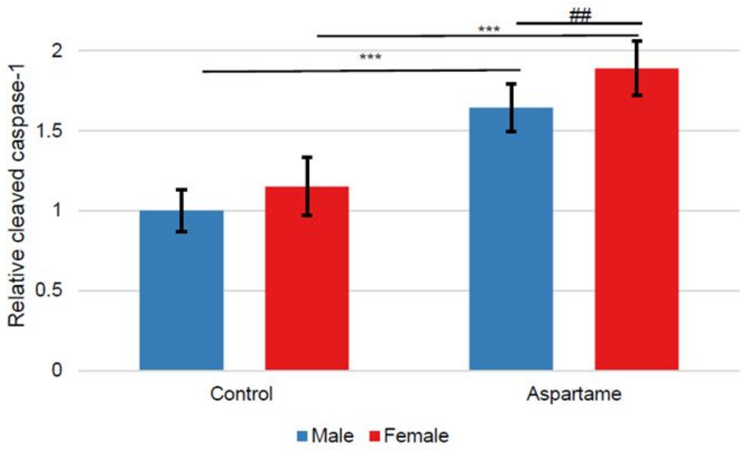

Figure S4.

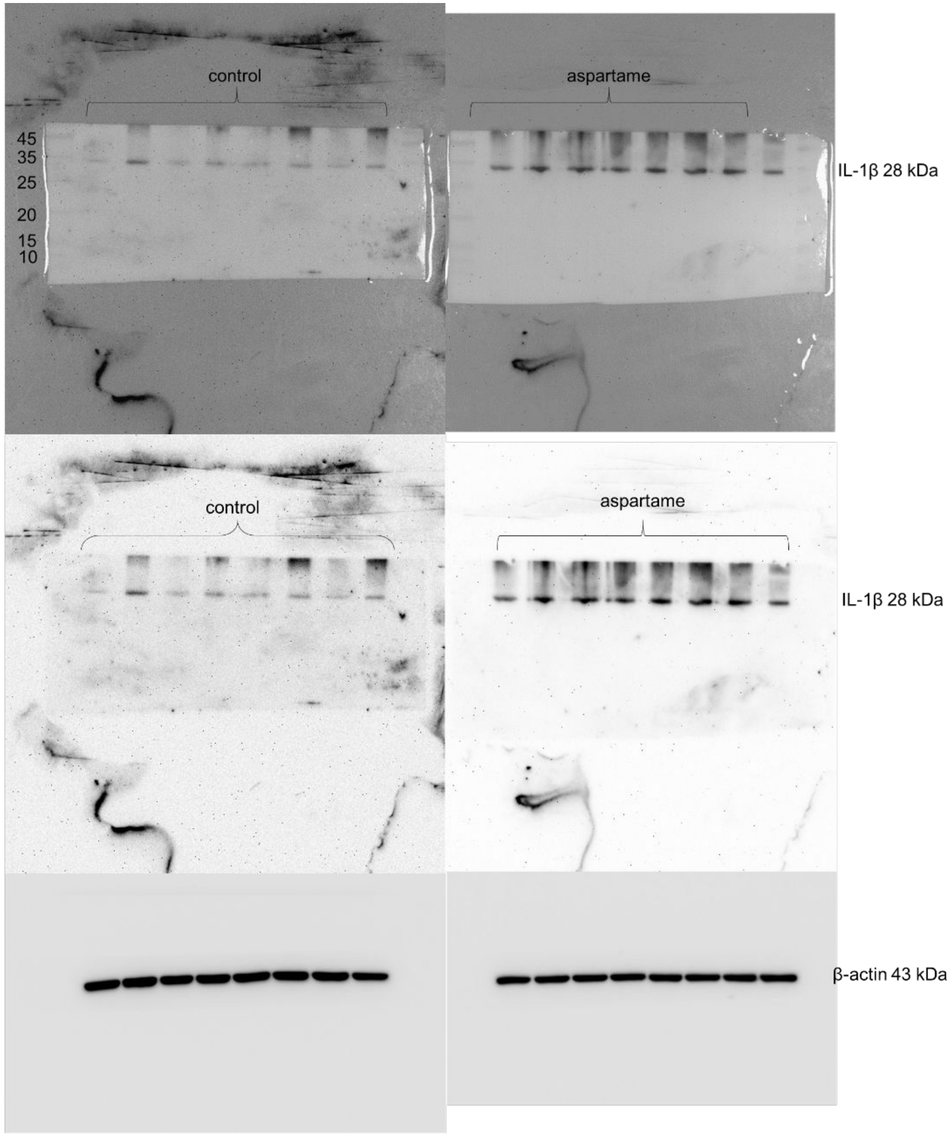
control
aspartame
45
35
25
20
15
10
IL-1β 28 kDa
control
aspartame
IL-1β 28 kDa
β-actin 43 kDa

Supplementary Figure Legend

Fig. S1. Sex-specific alterations in oxidative stress markers in the lungs of postnatal day 21 offspring following maternal aspartame exposure. Bar graphs depict levels of oxidative stress markers measured by ELISA in male (left) and female (right) offspring from control and aspartame-exposed groups. **A**. 8-hydroxy-2'-deoxyguanosine (8-OHdG; ng/mL), and **B**. glutathione (GSH; µg/mL). Data are presented as mean ± SD (n = 7 per group). $**p < 0.01$, $***p < 0.001$.

Fig. S2. ASC (Apoptosis-associated Speck-like protein containing a CARD) expression in lung tissues of P21 offspring following maternal aspartame exposure. Uncropped images are shown in the following order: (top) original SDS-PAGE gel with molecular weight markers, (middle) PVDF membrane probed for ASC (~22 kDa), and (bottom) PVDF membrane strip probed for β-actin (~43 kDa). The β-actin blot was obtained from the same membrane, which was cut prior to antibody incubation to reduce antibody use. Sample groups (control, aspartame) are labeled above the lanes.

Fig. S3. Cleaved caspase-1 expression in lung tissues of P21 offspring following maternal aspartame exposure. **A.** Quantification of cleaved caspase-1 levels in male (left) and female (right) offspring from control and aspartame-exposed groups (n = 4 per group). Data are shown as mean ± SD. $^{**}p < 0.01$, $^{***}p < 0.001$ vs. controls, determined by post hoc pairwise comparison following two-way ANOVA. **B.** Uncropped PVDF membrane showing cleaved caspase-1 (~20 kDa) and GAPDH (~36 kDa) bands from the same blot. Molecular weight markers are indicated on the left. Sample groups (control, aspartame) are labeled above the lanes. **C.** Direct comparison of male and female offspring on the same relative scale. Data are presented as mean ± SD. ## $p < 0.01$ female vs. male (averaged across exposure conditions).

Fig. S4. Interleukin-1β (IL-1β) expression in lung tissues of P21 offspring following maternal aspartame exposure. Uncropped images are shown in the following order: (top) original SDS-PAGE gel with molecular weight markers, (middle) PVDF membrane probed for IL-1β (~28 kDa), and (bottom) PVDF membrane strip probed for β-actin (~43 kDa). The β-actin blot was obtained from the same membrane, which was cut prior to antibody incubation. Sample groups (control, aspartame) are labeled above the lanes.

Supplementary Methods: aspartame dose calculation and human equivalent dose (HED) conversion

The aspartame dose of 0.25 g/L in drinking water was selected based on previous validation studies [16] and represents a high, yet physiologically relevant exposure level. The dose calculation and human equivalent conversion were performed as follows:

Mouse dose calculation:

- Estimated daily water intake: 4 mL per 25 g pregnant mouse
- Daily aspartame intake: (0.25 g/L × 4 mL) ÷ 25 g = 0.04 g/kg = 40 mg/kg body weight per day

HED Conversion:

HED conversion was performed using established allometric scaling based on body surface area:

- Formula: HED (mg/kg) = Animal Dose (mg/kg) × (Animal K_m / Human K_m)
- Mouse K_m = 3; Human K_m = 37 [17]
- HED = 40 mg/kg/day × (3/37) = 3.25 mg/kg/day

Human daily intake equivalent:

For an average 60 kg adult: 3.25 mg/kg/day × 60 kg = 195 mg/day

This corresponds to approximately one 355 mL can of diet soda (containing ~180-200 mg aspartame).

Regulatory context:

The selected dose therefore represents the upper range of typical human consumption, equivalent to ~1 can of diet soda per day for a 60 kg adult, while remaining well below the FDA acceptable daily intake (ADI) of 50 mg/kg/day. This framing positions our study as modeling high but physiologically relevant exposure conditions, suitable for evaluating potential developmental effects.
